# Trpm4 ion channels in pre-Bötzinger complex interneurons are essential for breathing motor pattern but not rhythm

**DOI:** 10.1371/journal.pbio.2006094

**Published:** 2019-02-21

**Authors:** Maria Cristina D. Picardo, Yae K. Sugimura, Kaitlyn E. Dorst, Prajkta S. Kallurkar, Victoria T. Akins, Xingru Ma, Ryoichi Teruyama, Romain Guinamard, Kaiwen Kam, Margaret S. Saha, Christopher A. Del Negro

**Affiliations:** 1 Department of Applied Science, Integrated Science Center, William & Mary, Williamsburg, Virginia, United States of America; 2 Biological Sciences, Louisiana State University, Baton Rouge, Louisiana, United States of America; 3 Signalisation, Electrophysiologie et Imagerie des Lésions d’Ischémie-Reperfusion Myocardique, Normandie Université, UNICAEN, Caen, France; 4 Department of Cell Biology and Anatomy, Chicago Medical School, Rosalind Franklin University, Chicago, Illinois, United States of America; 5 Department of Biology, Integrated Science Center, William & Mary, Williamsburg, Virginia, United States of America; Université de Montréal, Canada

## Abstract

Inspiratory breathing movements depend on pre-Bötzinger complex (preBötC) interneurons that express calcium (Ca^2+^)-activated nonselective cationic current (*I*_CAN_) to generate robust neural bursts. Hypothesized to be rhythmogenic, reducing *I*_CAN_ is predicted to slow down or stop breathing; its contributions to motor pattern would be reflected in the magnitude of movements (output). We tested the role(s) of *I*_CAN_ using reverse genetic techniques to diminish its putative ion channels Trpm4 or Trpc3 in preBötC neurons in vivo. Adult mice transduced with Trpm4-targeted short hairpin RNA (shRNA) progressively decreased the tidal volume of breaths yet surprisingly increased breathing frequency, often followed by gasping and fatal respiratory failure. Mice transduced with Trpc3-targeted shRNA survived with no changes in breathing. Patch-clamp and field recordings from the preBötC in mouse slices also showed an increase in the frequency and a decrease in the magnitude of preBötC neural bursts in the presence of Trpm4 antagonist 9-phenanthrol, whereas the Trpc3 antagonist pyrazole-3 (pyr-3) showed inconsistent effects on magnitude and no effect on frequency. These data suggest that Trpm4 mediates *I*_CAN_, whose influence on frequency contradicts a direct role in rhythm generation. We conclude that Trpm4-mediated *I*_CAN_ is indispensable for motor output but not the rhythmogenic core mechanism of the breathing central pattern generator.

## Author summary

Breathing behavior consists of periodic movements of the chest and airways that ventilate the lungs. The brain must generate a rhythm and form a motor output pattern to make breathing movements happen. Here, we address the ion channel–level neural origins of breathing, particularly the role of a class of transient receptor potential (Trp) channels hypothesized to be rhythmogenic. Using genetic techniques to knockdown these channels in a specialized brainstem site known for its respiratory rhythmogenic role, we surprisingly did not find the expected changes in breathing frequency. Rather, we measured progressive attenuation of breath magnitude, which in some cases led to fatal breathing pathologies. Therefore, the importance of this ion channel class is not respiratory rhythm generation per se but rather governing the motor output pattern. These results cause us to re-evaluate whether rhythm and pattern are discrete neural processes or instead inextricably linked in microcircuits of the central nervous system that generate and control motor behaviors.

## Introduction

Inspiratory breathing movements in mammals emanate from neural rhythms of the pre-Bötzinger complex (preBötC) in the lower brainstem [1–4]. If preBötC excitability is sufficiently high, then its constituent interneurons burst synchronously and drive motor output [5,6]. Calcium (Ca^2+^)-activated nonselective cation current (*I*_CAN_) may play a substantial role by generating the depolarization underlying inspiratory bursts, i.e., the inspiratory drive potential [7,8]. *I*_CAN_-mediated inspiratory drive potentials are evoked by excitatory synaptic inputs and intrinsic Ca^2+^ signaling in the context of rhythmic network activity [7–12], but whether inherently part of rhythmogenesis or effectuating motor pattern, the role(s) of *I*_CAN_ remain incompletely understood.

The ion channels that give rise to *I*_CAN_ have not been identified, but transient receptor potential (Trp) channels are likely candidates. There are 28 different Trp subtypes, subdivided into seven subfamilies, which broadly share structural similarity but vary in their permeability to mono- and divalent cations as well as their activation mechanisms. Widely expressed in the nervous system, Trp channels mediate phototransduction, thermosensation, gustation, nociception, and a range of homeostatic functions [13–16].

A limited subset of Trps from the Trpm (M for melastatin) and Trpc (C for canonical) subfamilies are implicated in respiration. We originally posited that either Trpm4 or Trpm5 (or both) gave rise to *I*_CAN_ in the preBötC [8,17] because they form flufenamic acid (FFA)-sensitive 24-picosiemens (pS) conductance monovalent cation channels, which are gated by intracellular Ca^2+^ transients and modulated by phosphoinositides [18–20]. However, 24-pS ion channel activity recorded in sync with fictive inspiration in preBötC neurons is sensitive to ATP^−4^ [21,22]. Trpm4, but not Trpm5, is sensitive to blockade by highly charged polyatomic anions like ATP^−4^ [23–26], which is only consistent with Trpm4 in the preBötC. As further confirmation that Trpm4, rather than Trpm5, is involved with respiratory rhythm and pattern, we recently showed that *Trpm4*, but not *Trpm5*, transcripts are expressed in excitatory preBötC neurons [27].

Trpc3 forms 23-pS conductance ion channels permeable to mono- and divalent cations [28,29], which seems inconsistent with the Ca^2+^-activated monovalent intrinsic current *I*_CAN_. Nevertheless, the heteromeric association of Trpc3 with Trpc1 markedly reduces Ca^2+^ permeability [30], and *Trpc1* transcripts are expressed at approximately the same level as *Trpm4* in the preBötC [27], so a heteromeric Trpc3-mediated monovalent *I*_CAN_ is feasible. Trpc3 has already been implicated in regulation of respiratory rhythm [31]. Furthermore, RNA sequencing (RNA-seq) revealed *Trpc3* to be the single most abundantly expressed *Trp* gene in rhythmogenic and nonrhythmogenic preBötC neurons [27], so it rises to the top of candidates from the Trpc family as an ion channel subunit that could mediate or contribute to *I*_CAN_.

Excitatory interneurons that form the inspiratory core oscillator in the preBötC are derived from precursors expressing the transcription factor developing brain homeobox 1 (Dbx1) [32–37]. A subset of Dbx1-derived neurons also shapes motor output pattern [38,39]. Whether for rhythm or pattern generation, Dbx1-derived neurons express *I*_CAN_ [33,40]. Neighboring non-Dbx1–derived interneurons, presumably inhibitory, influence breathing motor pattern and inspiratory–expiratory phase transition [41–44], so *I*_CAN_, which is ubiquitous in the preBötC [8], is relevant to their role(s) as well. Therefore, we evaluated the breathing-related role(s) of Trpm4 and Trpc3 as two likely candidates underlying *I*_CAN_ in preBötC neurons. Attenuating the activity of Trpm4, but much less so Trpc3, affects breathing behavior by attenuating motor output functions of the preBötC but not rhythm generation per se. Koizumi and colleagues reach a similar conclusion using an in vitro approach [45]. We propose that Trpm4 largely mediates *I*_CAN_, which forms a part of the pattern generator microcircuit rather than the core oscillator for breathing.

## Results

### Trpm4 and Trpc3 ion channels in preBötC interneurons

**Fig 1:**
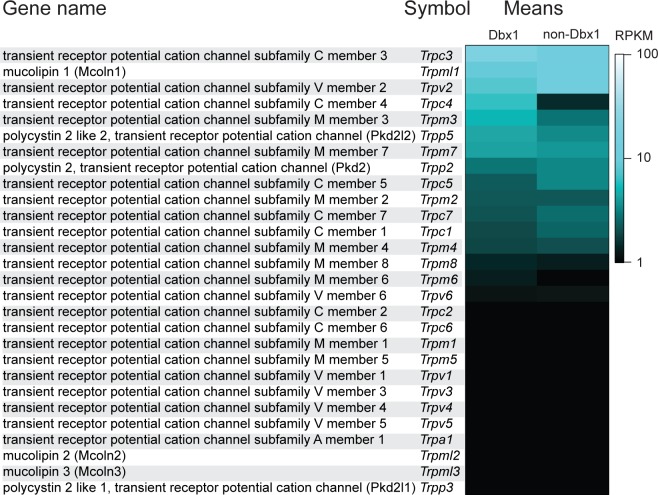
Quantification of *Trp* transcript expression in preBötC neurons. Mean *Trp* mRNA expression in reads per kilobase of transcript per million mapped reads (RPKM) in Dbx1 and non-Dbx1 preBötC neurons from Dbx1;Ai9 mice at postnatal day 2, pseudocolor scale at right. Transcripts organized by relative expression levels in the Dbx1 neurons. Some of the *Trp* transcript data were displayed in a different format as supplemental information in [27]. Data are publicly available in the NCBI Gene Expression Omnibus database, https://www.ncbi.nlm.nih.gov/geo/, accession no. GSE100356. Dbx1, developing brain homeobox 1; NCBI, National Center for Biotechnology Information; preBötC, pre-Bötzinger complex; RPKM, reads per kilobase of transcript per million mapped reads; Trp, transient receptor potential.

For reasons outlined in the Introduction, we considered Trpm4 or Trpc3 (or both) to be likely candidates that mediate *I*_CAN_. Having sequenced the transcriptome of Dbx1 and non-Dbx1 preBötC neurons [27] and released those data in the public domain (National Center for Biotechnology Information [NCBI] Gene Expression Omnibus database, accession no. GSE100356), here, we rank-ordered *Trp* transcripts according to expression level. Sixteen out of 28 *Trp* genes showed nonzero expression in the preBötC (Fig 1). *Trpc3* had the highest level of expression (reads per kilobase of transcript per million mapped reads [RPKM] 18.5 in Dbx1 neurons, 11.9 in non-Dbx1 neurons). *Trpm4* expression (RPKM 0.9 in Dbx1 neurons, 1.0 in non-Dbx1 neurons) was below the median RPKM for all expressed genes (1.7, [27]), but *Trpm4* expression was in the top half of all *Trp* channel genes and, for reasons outlined in the Introduction, was a strong candidate a priori for *I*_CAN_. Other Trp channels among the 16 we detected are unlikely to give rise to *I*_CAN_ for the following reasons. Trpm2, Trpm3, Trpm8, and Trpv2 are thermosensitive; Trpc1, Trpc4, Trpc5, and Trpc7 form store-operated channels (SOC); and Trpm7 and Trpv6 are primarily divalent ion channels [14,16]. Finally, the single-channel conductance of Trpp2 and Trpp5 exceed 100 pS [46], which is incompatible with 24-pS channel activity in sync with inspiration in preBötC neurons [21].

**Fig 2:**
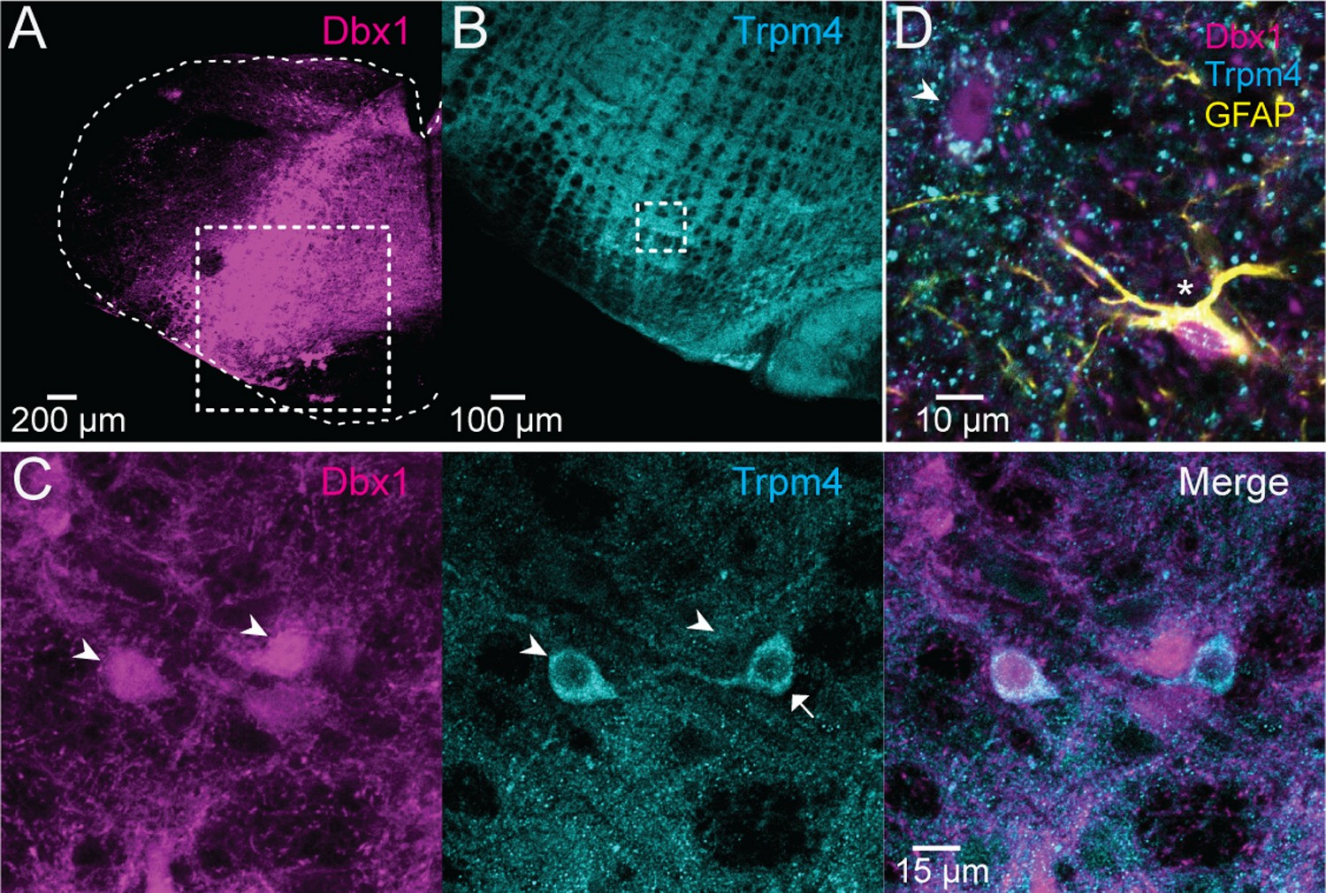
Trpm4 ion channels in preBötC neurons. (A) Transverse section from an adult Dbx1;Ai9 mouse showing tdTomato (magenta) expression in Dbx1-derived cells. (B) Selection box from A encompasses the preBötC and shows Trpm4 expression (cyan). (C) Selection box from the preBötC in B showing tdTomato (Dbx1) and Trpm4 expression in preBötC neurons. Trpm4 is expressed in both Dbx1 neurons (arrowheads) and non-Dbx1 neurons (arrow). (D) GFAP expression (yellow) identifies Dbx1-derived astrocytes of the preBötC (yellow and magenta, marked with an asterisk), which lack Trpm4 expression. Arrowhead identifies a Dbx1 neuron with Trpm4 expression. Separate scale bars are shown for A, B, C, and D. Dbx1, developing brain homeobox 1; GFAP, glial fibrillary acidic protein; preBötC, pre-Bötzinger complex; Trp, transient receptor potential.

**Fig 3:**
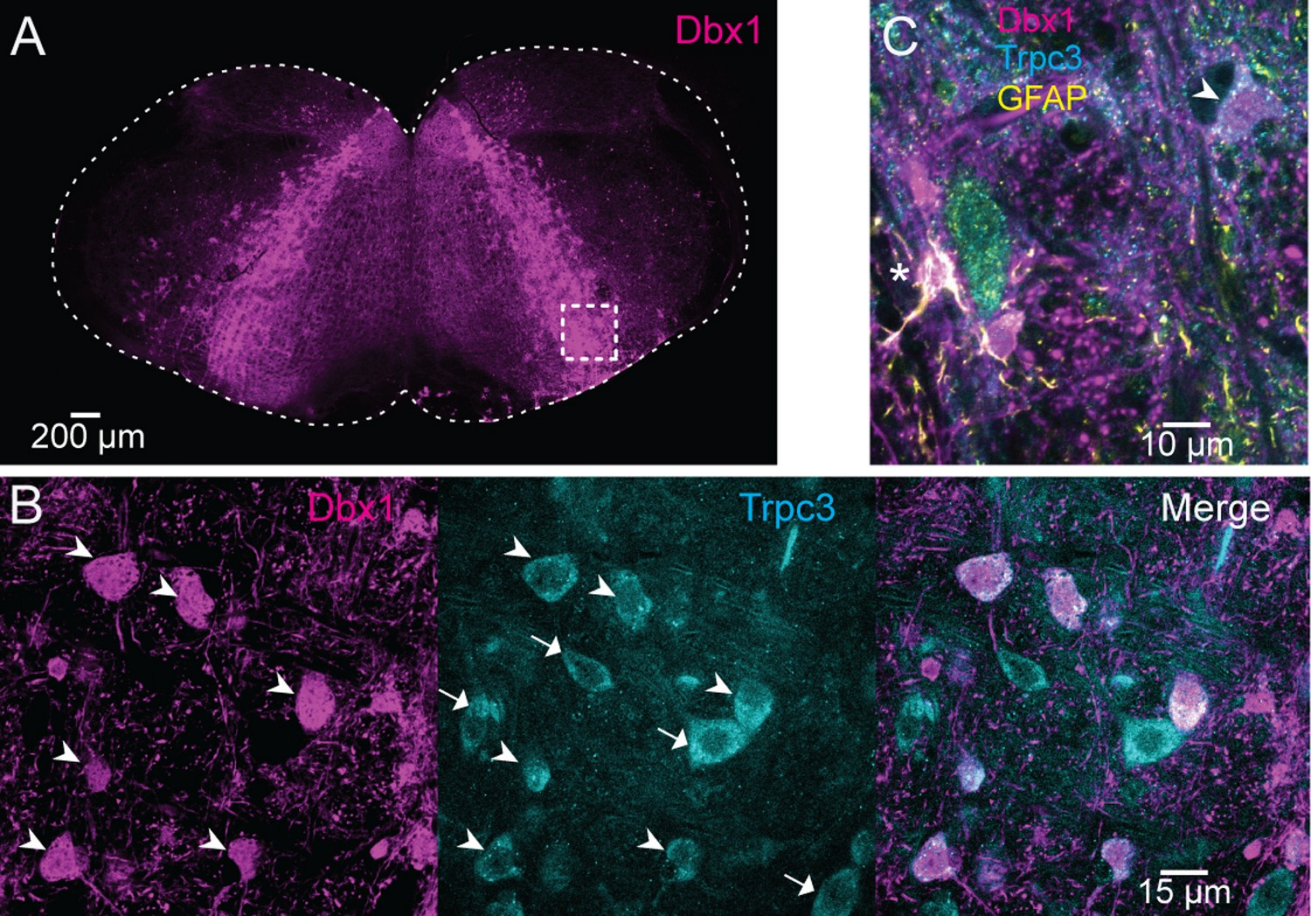
Trpc3 ion channels in preBötC neurons. (A) Transverse section from an adult Dbx1;Ai9 mouse showing tdTomato (magenta) expression in Dbx1-derived cells. (B) Selection box from A focuses on the preBötC core and shows tdTomato (Dbx1) and Trpc3 expression (cyan). Trpc3 is expressed in both Dbx1 neurons (arrowheads) and non-Dbx1 neurons (arrows). (C) GFAP expression (yellow) identifies Dbx1-derived astrocytes of the preBötC (yellow and magenta, marked with an asterisk), which lack Trpc3 expression. Arrowhead identifies a Dbx1 neuron with Trpc3 expression. Separate scale bars are shown for A, B, and C. Dbx1, developing brain homeobox 1; GFAP, glial fibrillary acidic protein; preBötC, pre-Bötzinger complex; Trp, transient receptor potential.

Having quantified *Trpm4* and *Trpc3* transcripts in Dbx1 (excitatory) and non-Dbx1 (presumably inhibitory) preBötC neurons through RNA-seq, we examined expression of these genes at the protein level via immunohistochemical labeling. In adult and neonatal mice, we detected Trpm4 (Figs 2 and S1) and Trpc3 (Figs 3 and S2) in both Dbx1-derived and non-Dbx1–derived preBötC interneurons, which suggests that these channels could comprise *I*_CAN_ and thus be relevant to breathing in adult as well as perinatal mice. Dbx1-derived cells in the preBötC include astrocytes [32,38,47], which did not express Trpm4 (Fig 2D) or Trpc3 (Fig 3C).

### shRNA knockdown of Trpm4 and Trpc3 affect breathing behavior

We used short hairpin RNA (shRNA) to knock down Trp channels in adult mice. Adeno-associated viruses (AAVs) encoding either a Trpm4- or Trpc3-targeted shRNA (hereafter referred to as Trpm4 or Trpc3 shRNA) as well as enhanced green fluorescent protein (eGFP) were injected in the preBötC. First, we injected shRNA unilaterally into the left preBötC in conjunction with contralateral (right side) injections of a nontargeting control sequence (*n* = 6 mice total). Five weeks later, *Trpm4* and *Trpc3* expression were 72% ± 3% and 76% ± 4% lower, respectively, in the left preBötC compared to the right preBötC, which shows that shRNAs effectively impede *Trp* ion channel gene expression in preBötC neurons (Table 1).

**Table 1:** Quantification of transcript copies of *Trp* genes per 2.5 ng cDNA synthesized from RNA extracted from the preBötC of adult mice and measured by ddPCR.

ddPCR target	Trp shRNA (left preBötC)	Nontargeting control seq. (right preBötC)	Expression change due to shRNA
*Trpm4*	12	48	75% decrease
*Trpm4*	13	47	72% decrease
*Trpm4*	12	61	80% decrease
*Trpc3*	111	395	72% decrease
*Trpc3*	120	387	69% decrease
*Trpc3*	106	419	75% decrease

**Abbreviations:** ddPCR, droplet digital PCR; preBötC, pre-Bötzinger complex; shRNA, short hairpin RNA; Trp, transient receptor potential.

We next assessed the time course of Trp channel knockdown by injecting another cohort of mice with shRNA and then measuring Trpm4 and Trpc3 protein expression in the preBötC over time (*n* = 12 mice injected with Trpm4 shRNA and *n* = 12 mice injected with Trpc3 shRNA). Western blots quantifying protein expression showed a monotonic decline in Trpm4 and Trpc3 ion channel expression as a function of number of days postinjection. Trpm4 was reduced by 12%, 42%, 62%, and 65% and Trpc3 was reduced by 33%, 38%, 52%, and 68% at days 10, 20, 30, and 40, respectively (S3 Fig).

#### Trpm4 shRNA

**Fig 4:**
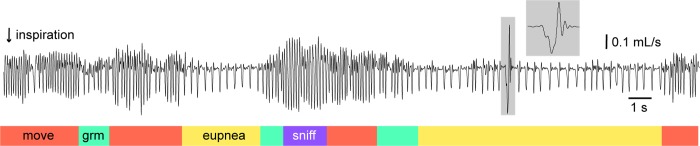
Breathing in an awake, intact adult mouse. A 30 sec continuous sample of breathing via whole-body plethysmography in a CD-1 mouse. Inspiratory airflow points down. The trace shows exploratory movements (orange), grooming (green), and high-frequency sniffing (purple), in addition to eupnea (yellow). One sigh (gray, expanded in the inset) characterized by 2-fold larger inspiration compared to a typical eupneic breath, followed by expiration. Time and airflow calibrations are shown. Raw data can be found in the Supporting information (S1 Data).

We measured breathing behavior intermittently in 11 awake adult mice for six weeks following bilateral injection of Trpm4 shRNA. Fig 4 shows typical preinjection behavior with bouts of eupnea (i.e., calm breathing), exploratory movements, grooming, and one sigh highlighted in gray (also shown in the inset at higher sweep speed). Baseline measurements of eupnea include tidal volume (V_T_) of 0.2 ± 0.04 ml, breathing frequency of 4.4 ± 1.2 Hz, inspiratory time (T_I_) of 161 ± 50 ms, and minute ventilation (MV) of 0.05 ± 0.01 l/min. The average baseline sigh frequency measured 0.60 ± 0.12 min^−1^.

**Fig 5:**
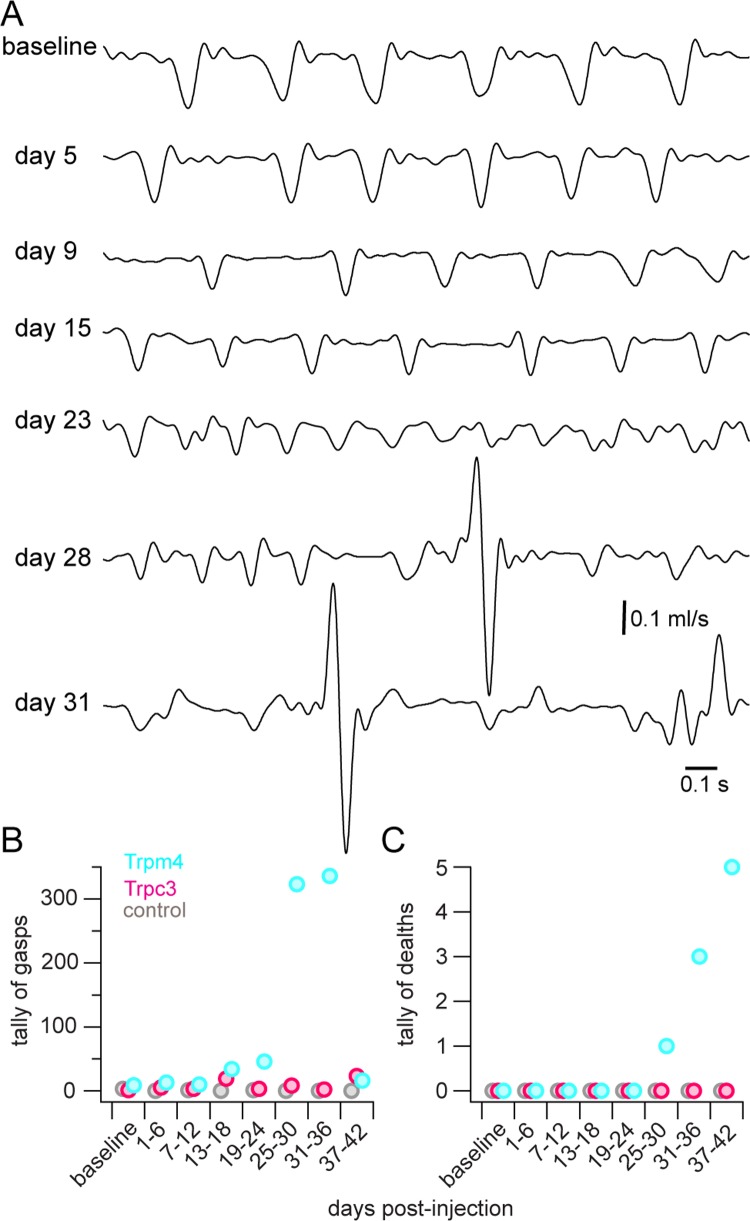
Longitudinal study of breathing in a Trpm4 shRNA-injected mouse. (A) Breathing at different time points from before injection (baseline) until day 31 after injection of Trpm4 shRNA in an adult CD-1 mouse. Gasps on days 28 and 31 are recognized by rapid active exhale (upward trajectory before inspiration), followed by inhale (downward trajectory) efforts. This mouse died on day 32. (B,C) Group data showing tallies of gasps (B) and deaths (C) in mice injected with Trpm4 (cyan), Trpc3 (magenta), and nontargeting control (gray) shRNA. Elapsed time following shRNA injection was binned in 6-day increments. Primary data can be found in the Supporting information (S1 Data). shRNA, short hairpin RNA; Trp, transient receptor potential.

Mice injected with Trpm4 shRNA showed a progressive diminution of V_T_ and T_I_, combined with an increase in frequency (Fig 5A). These changes were pathological. After day 13 of the observation period, six out of 11 animals were gasping at rates exceeding 0.2 min^−1^ (max rate 10.1 min^−1^, mean ± SD measured 1.5 ± 3.3 min^−1^) in normoxia, which was never observed in baseline conditions or prior to day 13 postinjection (Fig 5B tallies gasps in the cohort). Five out of the 11 animals apparently died of respiratory failure after 21–37 days (Fig 5C). In each case, we confirmed the expression of eGFP, i.e., Trpm4 shRNA, in the preBötC as well as the paucity of eGFP in adjacent respiratory sites, including the rostrally located Bötzinger complex of the medulla and the ventral respiratory group of the upper cervical spinal cord (S4 Fig).

**Fig 6:**
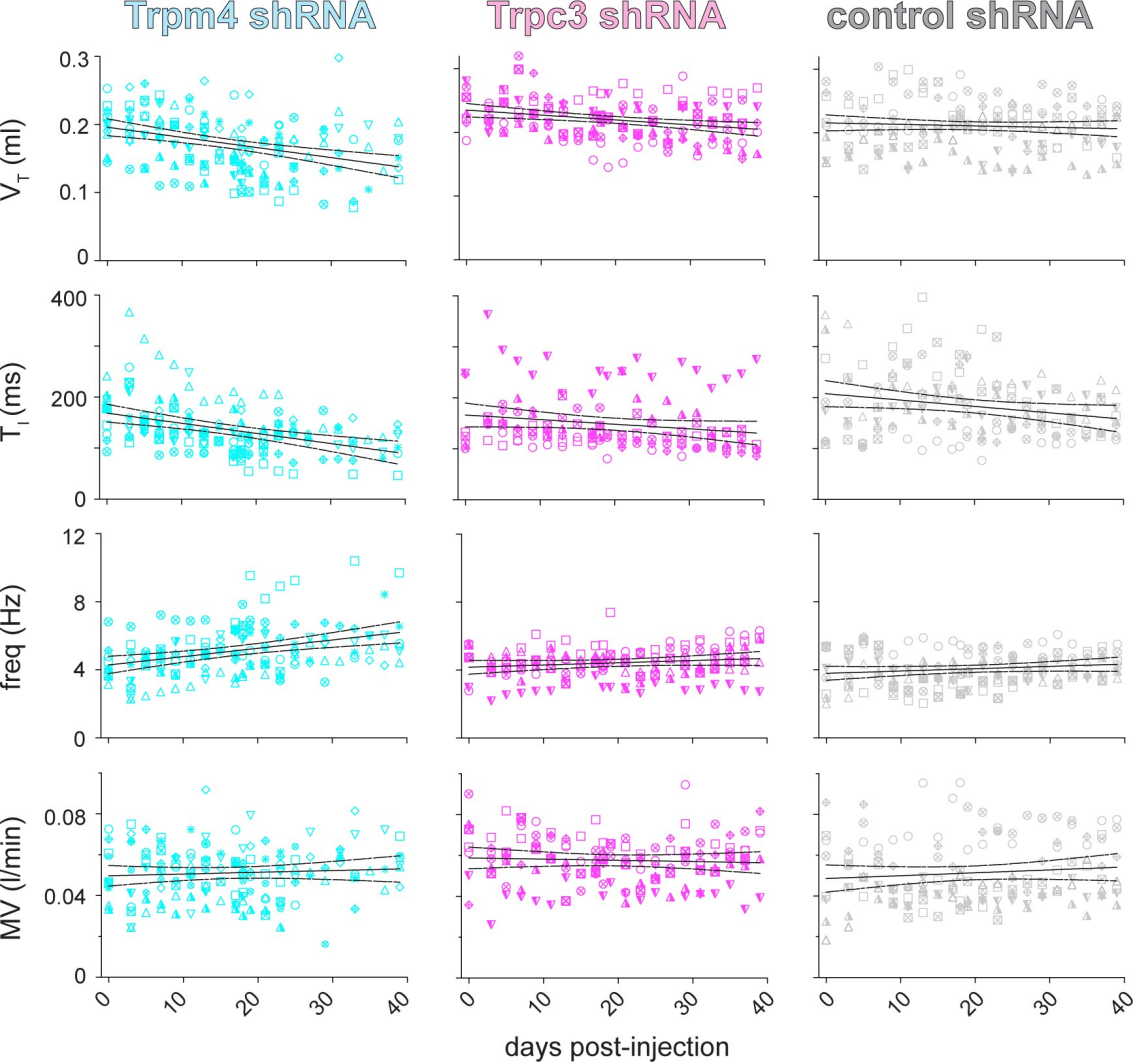
Longitudinal study of breathing after shRNA injections in three mouse cohorts. Breathing measurements in awake adult CD-1 mice after shRNA injection targeting Trpm4 (left, cyan), Trpc3 (middle, magenta), and a nontargeting control sequence (right, gray). Rows from top to bottom show V_T_, T_I_, frequency, and MV. Each animal was assigned a unique symbol (circle, square, triangle, hexagon, plus sign, asterisk, and variations on those symbols), which tracks the breathing measurements for that animal during the postinjection observation period. Best-fit lines and the 95% confidence intervals are shown. The number of points in the left column (Trpm4) decreases as animals died of respiratory failure starting at the bin for days 25–30. Primary data can be found in the Supporting information (S1 Data). shRNA, short hairpin RNA; Trp, transient receptor potential.

We applied a linear regression model to analyze how Trpm4 shRNA affected breathing measurements over time (Fig 6). The slope of V_T_ versus day post-shRNA was significantly nonzero (Fig 6, 1st row, cyan, r^2^ = 0.12, *P* = 1E-5), emphasizing a significant progressive diminution of V_T_ during the observation period. V_T_ decreased 31% to 0.13 ± 0.03 ml by the endpoint of the experiment (*n* = 11). Note that endpoint V_T_ was computed using either the V_T_ at the end of 6 weeks (*n* = 6) or on the last measurement day prior to death (*n* = 5).

The slope of T_I_ versus day post-shRNA was also significantly nonzero (Fig 6, 2nd row, cyan, r^2^ = 0.2, *P* = 4E-8). The endpoint T_I_ decreased 28% to 111 ± 37 ms (*n* = 11). The slope of frequency versus day post-shRNA was significantly nonzero (Fig 6, 3rd row, cyan, r^2^ = 0.14, *P* = 1.2E-6); in this case, slope was positive. The endpoint frequency increased 35% to 5.6 ± 1.9 Hz (*n* = 11). In contrast, MV did not change during the observation period because decreases in breath magnitude, quantified by V_T_ and T_I_, were counterbalanced by frequency increases (Fig 6, 4th row, i.e., the slope of MV versus day post-shRNA was ostensibly zero, r^2^ = 0.004, *P* = 0.5). The endpoint MV measured 0.05 ± 0.02 l/min (*n* = 11). Sigh frequency significantly decreased following Trpm4 shRNA; by the endpoint, it was 30% lower, 0.42 ± 0.18 min^−1^ (*P* = 0.049).

#### Trpc3 shRNA

Baseline measurements of eupnea for Trpc3 shRNA-injected mice (*n* = 7) include V_T_ of 0.24 ± 0.03 ml, T_I_ of 153 ± 65 ms, frequency of 4.5 ± 1.2 Hz, and MV of 0.06 ± 0.02 l/min. The average baseline sigh frequency measured 0.60 ± 0.05 min^−1^.

Again, we applied linear regression to analyze breathing as a function of elapsed time following Trpc3 shRNA injection. V_T_ and T_I_ decreased progressively (Fig 6, 1st and 2nd rows, magenta; V_T_: r^2^ = 0.07, *P* = 0.001; T_I_: r^2^ = 0.04, *P* = 0.02). The endpoint V_T_ decreased by 13% to 0.21 ± 0.03 ml, and the endpoint T_I_ decreased by 14% to 132 ± 56 ms. However, the low values of r^2^ (0.07 and 0.04, respectively) and the small total changes in V_T_ and T_I_ (13% and 14%, respectively) diminish the physiological significance of these results, even though they pass the threshold for statistical significance. The changes in V_T_ and T_I_ were less substantial in Trpc3 shRNA-injected mice compared to Trpm4 shRNA-injected mice: a 13% versus 31% decrease in V_T_ and a 14% versus 28% decrease in T_I_, respectively.

Frequency did not change as a function of day post-shRNA (Fig 6, 3rd row, magenta, r^2^ = 0.03, *P* = 0.06); its endpoint measured 4.9 ± 1.1 Hz. Neither did MV change during the observation period (Fig 6, 4th row, magenta, r^2^ = 0.003, *P* = 0.5). Endpoint MV measured 0.06 ± 0.01 l/min. Sigh frequency was also unchanged, measuring 0.60 ± 0.18 min^−1^ (*P* = 0.37) at the endpoint.

Four of the seven Trpc3 shRNA-injected mice gasped at rates exceeding 0.2 min^−1^ at some point during the observation period, but the tally of gasps in this cohort never exceeded 23, whereas the gasp tally in Trpm4 shRNA-injected mice exceeded 300 after 30 days of observation (Fig 5B). All seven Trpc3 shRNA-injected mice were alive after 6 weeks.

#### Nontargeting shRNA

Baseline measurements of eupnea for nontargeting control shRNA-injected mice (*n* = 8) include V_T_ of 0.22 ± 0.05 ml, frequency of 3.8 ± 1.4 Hz, T_I_ of 209 ± 102 ms, and MV of 0.05 ± 0.02 l/min. The average baseline sigh frequency measured 0.54 ± 0.48 min^−1^.

Mice injected with nontargeting shRNA (*n* = 8) showed no changes in V_T_, frequency, MV, or sigh frequency during the observation period. V_T_ measured 0.21 ± 0.03 ml, frequency measured 4.3 ± 0.7 Hz, and MV measured 0.05 ± 0.01 l/min at the endpoint (Fig 6, 1st, 3rd, and 4th rows, gray; V_T_: r^2^ = 0.005, *P* = 0.4; frequency: r^2^ = 0.025, *P* = 0.053; MV: r^2^ = 0.01, *P* = 0.2). Sigh frequency measured 0.47 ± 0.12 min^−1^ (*P* = 0.27) at the endpoint.

We observed a significantly nonzero slope of T_I_ versus day post-shRNA (Fig 6, 2nd row, gray, r^2^ = 0.05, *P* = 0.005). T_I_ measured 160 ± 33 ms at the endpoint, a 23% decrease that we conclude is not physiologically meaningful because there was no corresponding change in V_T_. No control animals gasped at any stage and all eight mice were alive 6 weeks after shRNA injection.

### Trpm4 and Trpc3 influence preBötC bursts and rhythm in vitro

#### preBötC field recordings

**Fig 7:**
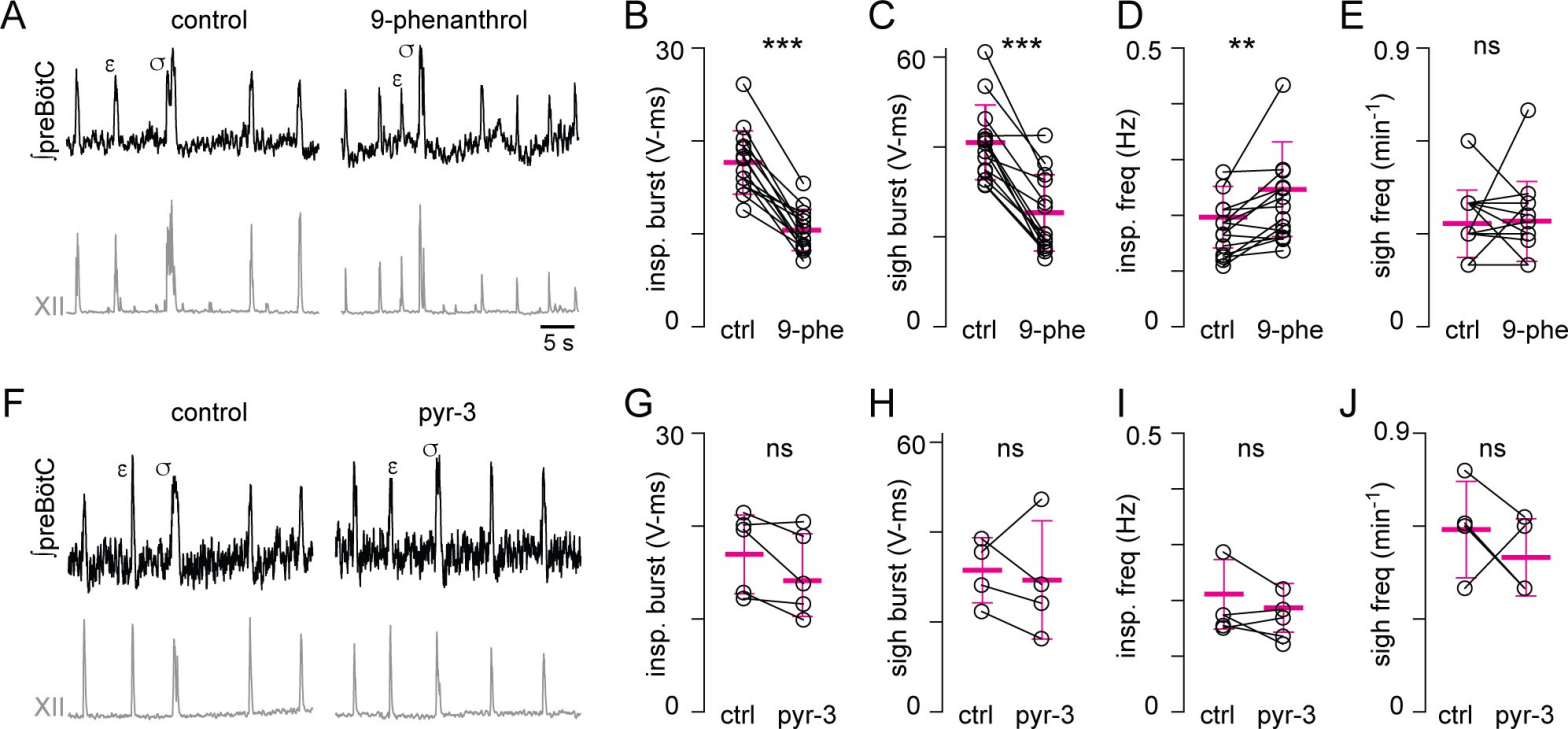
Trpm4 but not Trpc3 antagonists attenuate preBötC rhythms in vitro. (A) preBötC field potentials (black) and XII output (gray) in control and at steady state following approximately 20 min application of 100 μM 9-phenanthrol in neonatal CD-1 mice. A typical eupnea-related inspiratory burst is marked with ε; a typical sigh burst is marked with σ. (B–E) Area of inspiratory (B) and sigh (C) bursts as well as inspiratory (D) and sigh (E) frequency in control and 9-phenanthrol. (F) preBötC field potentials (black) and XII output (gray) in control and at steady state following approximately 20 min application of 10 μM pyr-3. Again, ε marks a eupnea-related inspiratory burst; σ marks a sigh burst. (G–J) Area of inspiratory (G) and sigh (H) bursts as well as inspiratory (I) and sigh (J) frequency in control and pyr-3. Mean ± SD shown (magenta) for all graphs in B–D and G–J. Primary data can be found in the Supporting information (S1 Data). preBötC, pre-Bötzinger complex; pyr-3, pyrazole-3; XII, hypoglossal nerve root.

We tested the effect of the Trpm4 antagonist 9-phenanthrol and Trpc3 antagonist pyrazole-3 (pyr-3) (in separate experiments) on the magnitude of rhythmic preBötC field potentials in a slice model of breathing. The preBötC in vitro generates eupnea-related inspiratory bursts, which typically occur at approximately 0.2 Hz, as well as larger magnitude sigh-related bursts that occur at 0.3–0.6 min^−1^ and for which each sigh burst is followed by a prolonged pause [48,49]. The half-maximal effective concentration (EC_50_) for 9-phenanthrol in the preBötC measured 32 µM (S5 Fig), and it is typically applied to excitable cells at a dose of 100 µM [50]. Exposing slices to 9-phenanthrol significantly reduced the area of the preBötC inspiratory bursts by 42% (*P* = 4E-7) and sigh bursts by 39% (*P* = 4E-6), with a 26% increase in inspiratory frequency (*P* = 0.006) (Fig 7A–7E, all *n* = 14) but no significant change in sigh frequency (*P* = 0.9). These data suggest that Trpm4 influences inspiratory and sigh burst area in the preBötC and, further, that Trpm4 attenuation increases inspiratory frequency, but not sigh frequency, in vitro.

We could not determine the EC_50_ for pyr-3 in the preBötC because of its inconsistent effects on respiratory network activity in vitro (S5 Fig). Therefore, we applied pyr-3 at 10 μM, consistent with the biophysics literature [51,52]. Pyr-3 had no effect on any measurement; neither the area nor the frequency of preBötC inspiratory (*n* = 5) and sigh (*n* = 4) bursts registered any change (Fig 7F–7J, inspiratory bursts [*P* = 0.1], sigh bursts [*P* = 0.7], inspiratory frequency [*P* = 0.3], sigh frequency [*P* = 0.4]). These data suggest that Trpc3 has little influence on inspiratory or sigh burst generation considering collective activity among all preBötC neurons and that Trpc3 does not influence the frequency of inspiration or sighs in vitro.

#### Whole-cell recordings from Dbx1 preBötC neurons

**Fig 8:**
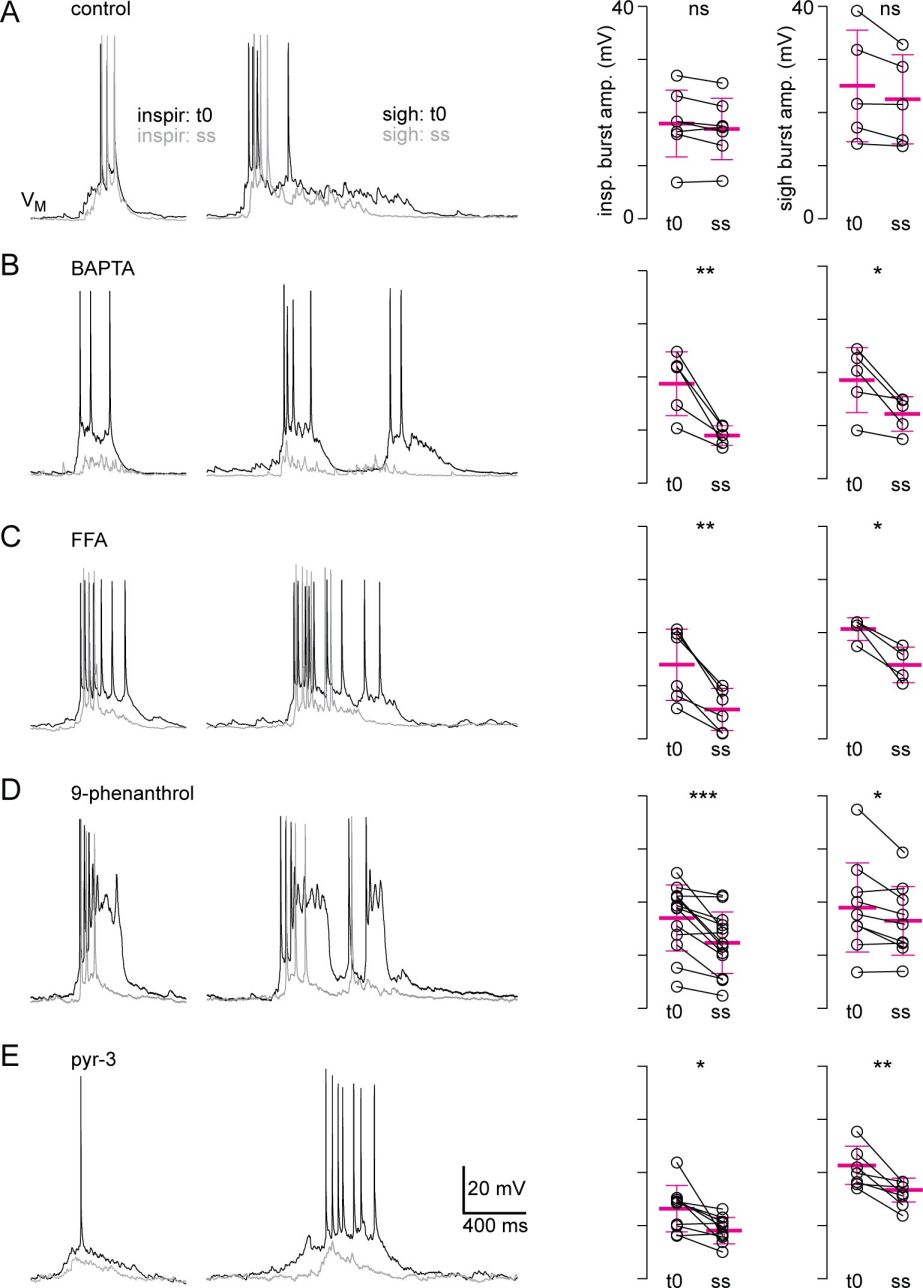
Trpm4 and Trpc3 antagonists attenuate inspiratory and sigh bursts of Dbx1 preBötC neurons. Data are from neonatal Dbx1;Ai9 mice. Each row shows inspiratory (left) and sigh bursts (right) superimposed at the onset of whole-cell recording (t0, black) and at steady-state (ss, gray, following approximately 20 min of whole-cell dialysis or [in the case of pyr-3] bath application). Group data plotted at far right with means and SD bars (magenta). (A) no drug dialysis, (B) BAPTA, (C) FFA, (D) 9-phenanthrol, and (E) pyr-3. Primary data can be found in the Supporting information (S1 Data). BAPTA, (1,2-bis(2-aminophenoxy)ethane-*N*,*N*,*N′*,*N′*-tetraacetic acid); Dbx1, developing brain homeobox 1; FFA, flufenamic acid; preBötC, pre-Bötzinger complex; pyr-3, pyrazole-3; Trp, transient receptor potential.

Next, we evaluated the Trpm4 and Trpc3 contributions to *I*_CAN_-mediated inspiratory drive potentials and sigh bursts via individual Dbx1 preBötC neuron recordings in brainstem slices. We employed (whenever possible) intracellular pharmacology applied via the patch pipette to avoid network effects of the ion channel antagonists, which, if bath-applied, could diminish endogenous rhythmic drive to preBötC neurons and impact frequency (this concern pertains to 9-phenanthrol, given the network effects on burst area and frequency quantified in Fig 7). Control whole-cell recordings (without drug dialysis) exhibited no change in the amplitude of inspiratory drive potentials (*n* = 7) or sigh bursts (*n* = 5) during 40 min of continuous recording (Figs 8A and S6; inspiratory bursts *P* = 0.06, sigh bursts *P* = 0.09). Therefore, drug-induced changes in subsequent experiments cannot be attributed to rundown.

Inspiratory drive potentials were attenuated by 49% ± 13% following whole-cell dialysis of (1,2-bis(2-aminophenoxy)ethane-*N*,*N*,*N′*,*N′*-tetraacetic acid) (BAPTA) (Figs 8B and S6, *P* = 0.01, *n* = 5), which precludes *I*_CAN_ activation, and by 64% ± 16% following whole-cell dialysis of the general *I*_CAN_ antagonist FFA (Figs 8C and S6; *P* = 0.002, *n* = 6). Similarly, the amplitude of sigh bursts decreased 31% ± 17% in the presence of BAPTA (Figs 8B and S6; *P* = 0.03, *n* = 5) and decreased 33% ± 13% in the presence of FFA (Figs 8C and S6; *P* = 0.02, *n* = 4). These data re-emphasize that *I*_CAN_ contributes substantially to inspiratory bursts and further show that *I*_CAN_ contributes to sigh bursts, which has not been previously demonstrated. Intracellular dialysis of the Trpm4 antagonist 9-phenanthrol reduced the amplitude of inspiratory drive potentials by 28% ± 19% (*P* = 0.008, *n* = 13) and sigh bursts by 12% ± 12% (Figs 8D and S6, *P* = 0.027, *n* = 9). These data indicate that Trpm4 contributes to *I*_CAN_, as measured in field recordings and whole-cell recordings of Dbx1 preBötC neurons.

Pyr-3 is not effective when applied intracellularly [51] so that drug was added to the bath, which reduced the amplitude of inspiratory drive potentials by 26% ± 22% (*P* = 0.02, *n* = 10) and sigh bursts by 22% ± 13% (Figs 8E and S6; *P* = 0.009, *n* = 7). These data show that Trpc3 also influences the magnitude of inspiratory and sigh bursts in Dbx1-derived preBötC neurons and thus could contribute to *I*_CAN_ in core preBötC neurons.

9-phenanthrol had the same effect in field and Dbx1 whole-cell recordings, i.e., it diminished inspiratory and sigh bursts, but pyr-3 did not; it reduced inspiratory and sigh bursts in Dbx1 whole-cell recordings but not in preBötC field recordings. To examine a cellular basis for this disparity, we tested how Trp channel antagonists affect inhibitory (glycinergic) preBötC neurons visualized using mice that express eGFP under the control of the promotor of the glycine transporter 2 (GlyT2) gene, that is, GlyT2-eGFP mice [53]. Neither 9-phenanthrol nor pyr-3 had any measurable effect on inspiratory or sigh bursts (S7 Fig). Roughly equally divided into excitatory (Dbx1) and inhibitory (principally glycinergic [41,54]) interneurons, the negligible effect of pyr-3 in preBötC field recordings (Fig 7F–7J) could be attributed to its lack of effect in the population of glycinergic preBötC interneurons (S7 Fig).

## Discussion

In air-breathing mammals, humans among them, inspiration is the foremost motor phase that organizes the breathing cycle. It emerges from a network oscillator in the brainstem preBötC wherein excitatory synaptic interactions are rhythmogenic [1,3,55]. However, the role of cellular burst–generating intrinsic currents remains unresolved.

preBötC activity typically culminates with a robust inspiratory burst, whose underlying drive potential of 20–40 mV amplitude depends on synaptically evoked *I*_CAN_ [8,11,12,21,56]. Although it has previously been considered an integral part of the rhythm-generating mechanism [57,58], our data instead show that *I*_CAN_, mediated by Trpm4 (and possibly Trpc3 and other) ion channels, selectively influences the magnitude of preBötC output and breath size, and thus, its role pertains to the first stage of motor pattern generation rather than rhythmogenesis per se.

### Trpm4 mediates *I*_CAN_ in preBötC interneurons

Whereas 16 of 28 Trp channel genes are expressed in the preBötC, we favor Trpm4 as one major contributor to *I*_CAN_; Trpc3 appears to contribute as well. The other 14 Trps may give rise to ion channels that modulate preBötC activity or underlie cell signaling pathways but, for reasons argued above (see Results: Trpm4 and Trpc3 ion channels in preBötC neurons), are unlikely to mediate *I*_CAN_, whose phasic activation coincides with (probably to amplify) inspiration and sighs.

*I*_CAN_ was originally attributed to Trpm4 and Trpm5 because of their similar gating mechanisms, 24-pS single-channel conductance, phosphoinositide modulation, and pharmacology [8,25]. However, we can now rule out Trpm5 entirely because inspiratory phasic ion channel activity in preBötC neurons is ATP sensitive [21,22], which is a uniquely Trpm4 property [23–26], and no *Trpm5* transcripts are detectable in preBötC neurons (Fig 1 and [27]). Prior reports of Trpm5 in the preBötC [9,17] may reflect its expression in laryngeal motoneurons or preganglionic autonomic neurons of the nucleus ambiguus [59], which adjoin and partially overlap with the preBötC, as well as in non-neural tissues.

Trpc3 ion channels may also contribute to preBötC *I*_CAN_, given the preeminent expression level of *Trpc3* transcripts in Dbx1 and non-Dbx1 neurons (Fig 1 and [27]), 23-pS single-channel conductance [29,60], and phosphoinositide modulation [20,61,62]. However, Trpc3 Ca^2+^ permeability exceeds that of monovalent cations, i.e., P_Ca_/P_Na_ > 1 [14,16,46,63], which is inconsistent with a current like *I*_CAN_ that is selective for monovalent cations yet gated by Ca^2+^. Nevertheless, heteromeric Trpc channels that include Trpc1 diminish Ca^2+^ permeability such that P_Ca_/P_Na_ < 1 [30,64,65]. Trpc3 can form heteromeric channels with Trpc1 [65–68] as well as Trpc6 and Trpc7 [68,69]. *Trpc1* and *Trpc7* are expressed at approximately the same level as *Trpm4* in the preBötC, whereas *Trpc6* expression is near zero [27]. Therefore, given that Trpc3 and Trpc7 regulate respiratory rhythm [31,70], heteromeric ion channels in which Trpc3 associates with Trpc1 or Trpc7 are feasible and could contribute to *I*_CAN_.

Trpm4 and Trpc3 can form heteromeric ion channels in HEK293T cells, which modifies gating properties and Ca^2+^ permeability [71]. Although heteromeric ion channels that cross Trp subfamily boundaries have not been characterized in cells that natively express both subunits like preBötC neurons, the existence of Trpc3/Trpm4-mediated *I*_CAN_ is conceivable. However, given the disparate results following selective attenuation of Trpm4 and Trpc3 in vitro and in vivo, it seems more likely that homomeric Trpm4 and heteromeric Trpc3 channels contribute to separate ion channel populations whose aggregate represents whole-cell *I*_CAN_. Among these two channels, Trpm4 is predominant because the breathing phenotype was far stronger, and often fatal, in Trpm4 shRNA- compared to Trpc3 shRNA-injected mice. In vitro, preBötC field recordings only implicate Trpm4 because its antagonist 9-phenanthrol diminished preBötC inspiratory and sigh bursts, whereas the Trpc3 antagonist pyr-3 did not.

Nevertheless, in Dbx1 preBötC neurons, both Trpm4 and Trpc3 contributed to inspiratory and sigh bursts; pyr-3 and 9-phenanthrol nearly equivalently diminished inspiratory bursts, and pyr-3 reduced sigh bursts more than 9-phenanthrol. At face value, those data suggest that Trpc3 and Trpm4 contribute commensurately to burst generation in Dbx1 preBötC neurons, but we argue that because pyr-3 had to be bath-applied during whole-cell recordings, its effects were magnified by impacting all constituent Dbx1 neurons whose collective synaptic drive would have been diminished. 9-phenanthrol, by contrast, acted only through the patch pipette of the neuron being recorded, so network synaptic drive would not have been affected. Therefore, one cannot compare the relative pharmacological attenuation of drive potentials recorded in whole-cell conditions to reach a conclusion about which ion channel predominantly comprises *I*_CAN_; instead, we rely on the field recordings in vitro and shRNA experiments in vivo to evaluate their relative contributions.

We conclude that Trpm4 is the predominant ion channel underlying *I*_CAN_, a ubiquitous current in preBötC neurons. In Dbx1-derived preBötC neurons specifically, Trpm4 and Trpc3 may constitute *I*_CAN_, probably via separate ion channel populations, but the contribution of Trpm4 exceeds that of Trpc3.

### *I*_CAN_ is not rhythmogenic but critical for motor pattern

Slices that include the preBötC and hypoglossal nerve root (XII) nucleus retain the minimal microcircuit to generate fictive inspiration and sighs and thus are experimentally advantageous for studies of rhythm and pattern generation [72–75]. A rhythmogenesis model we promoted [58] posits that recurrent excitation triggers *I*_CAN_ to produce robust inspiratory bursts. That model predicts that *I*_CAN_ attenuation should either slow down the rhythm—because it would take longer for recurrent excitation to build up the inward currents needed to generate inspiratory bursts—or stop the rhythm entirely if recurrent excitation is insufficient to culminate the respiratory cycle. However, rhythms in vitro sped up after blocking Trpm4 (and did not change after blocking Trpc3). We conclude that *I*_CAN_ is not rhythmogenic, but what can explain the counterintuitive frequency increase? Inspiratory cycles in vitro are unconstrained by phasic synaptic inhibition from pulmonary stretch receptors in the periphery that ordinarily truncate inspiration and thus cut down on cycle time and increase frequency [42,43]. Removing that sensory feedback amplifies inspiratory bursts, which are followed by a pronounced refractory period that depresses frequency [42,76]. (Temperature differences also influence frequencies in vivo versus in vitro, but we refer the reader to [77] and instead focus on intrinsic and synaptic factors.) Here, pharmacological attenuation of Trpm4-dominated *I*_CAN_ also truncates inspiratory bursts, which produces the same net effect as sensorimotor feedback inhibition in vivo, namely increasing frequency.

Regarding pattern, *I*_CAN_ attenuation reduced drive potentials in Dbx1 preBötC neurons (which reflects Trpm4 and Trpc3 contributions). Trpm4-dominated *I*_CAN_ attenuation reduced preBötC field potentials for inspiration and sighs. Therefore, *I*_CAN_ is relevant because it governs preBötC burst magnitude and thus the ability to propagate inspiratory phase activity (and sighs) to premotor neurons and drive motor output. Our data and conclusions regarding inspiratory burst magnitude complement a recent report by Koizumi and colleagues [45], but we uniquely report the role of *I*_CAN_ in sighs in vitro.

We additionally knocked down Trpm4 and Trpc3 expression in the adult mouse preBötC. Attenuation of Trpm4-dominated *I*_CAN_ progressively decreased breath size (V_T_ and T_I_)—which is consistent with its role governing motor pattern identified in vitro—but also increased breathing frequency. That frequency effect in vivo may be partly attributable to decreasing the refractory period following inspiration in core preBötC neurons, as explained above. A more important factor may be that sentient mice with intact chemosensory feedback increased their breathing rate, either via increased respiratory drive or volitionally, because smaller breaths induced by channel knockdown—at the original frequency—were insufficient to meet oxygen demand. Recall that MV (the product of V_T_ and frequency) did not change prior to gasping and/or death, so the Trpm4 shRNA-injected mice were able to sustain ventilation via this compensatory mechanism for approximately 22 days.

Whereas inspiratory frequency increased following Trpm4 shRNA, we note that sigh rate decreased, and that may have further contributed to respiratory insufficiency by gradually diminishing the gas exchange surface area of the alveoli [1,74].

An increase in fictive (inspiratory) breathing frequency was also observed in brainstem-spinal cord preparations in situ following bath application of the Trpm4 antagonist 9-phenanthrol [45]. That frequency effect in situ is attributable (at least in part) to a different mechanism involving inhibitory microcircuits unrelated to sensorimotor integration, and extrinsic to the preBötC, that interact with preBötC excitatory microcircuits [42,78]. Those inhibitory circuits are excluded from slices but retained in in situ preparations. Trpm4 channels are expressed throughout the medulla and bath-applied 9-phenanthrol acts on the medulla in its entirety. Therefore, as 9-phenanthrol reduces postinspiration (i.e., the inspiratory–expiratory phase transition), it increases the inspiratory frequency in situ partly because of effects on inhibitory interneurons distributed throughout the medulla [45]. Whether Trpm4 channels are expressed specifically in the postinspiratory complex (PiCo, [79]) is not yet known, so PiCo-related contributions to frequency control in the Koizumi and colleagues [45] pharmacological experiments in situ cannot yet be evaluated. Here, we limited shRNA injection to the preBötC so the inhibitory microcircuits that govern postinspiration and inspiratory–expiratory phase transition, predominantly in the rostral medulla [42,79–81], would not be affected in our experiments in vivo.

Regarding motor output, inspiratory rhythm propagates caudally from the preBötC to phrenic premotor neurons of the rostral ventral respiratory group (rVRG) [39,82–84] and then to diaphragmatic phrenic motoneurons in the cervical spinal cord. If either Trpm4 or Trpc3 shRNA affected rVRG premotor neurons, then that could conceivably diminish breath size. However, posthoc histology showed a dearth of eGFP expression in the rVRG, a site that extends notably for approximately 2 mm in the anterior–posterior axis and is not limited to the immediate caudal vicinity of the preBötC. We conclude that is very unlikely that Trpm4 shRNA directly impacted diaphragmatic premotor neurons.

It is similarly unlikely that Trpm4 shRNA affected respiratory interneurons of the rostrally sited Bötzinger complex based on the low eGFP expression observed in that region. Nevertheless, because Bötzinger neurons are inhibitory [85–89], if Trpm4 shRNA had expressed there, it would disinhibit phrenic premotor neurons and phrenic motor neurons and thus probably augment the drive to the breathing pump rather than diminish it.

We observed eGFP expression dorsal to the preBötC, where airway premotor neurons and laryngeal motoneurons are located [38,90–94]. Therefore, Trpm4 knockdown could have affected airway patency but those airway effects would not impact tidal volume or inspiratory time, which are instead controlled by inspiratory pump musculature (e.g., diaphragm, external intercostals).

Attenuating Trpc3 (minimally) reduced breath size in vivo and in inspiratory drive potentials in Dbx1 preBötC neurons but did not affect preBötC field recordings. Only 35% of inhibitory (non-Dbx1) preBötC neurons express Trpc3 [45]. Furthermore, we measured no attenuation of inspiratory or sigh bursts in glycinergic preBötC neurons. Therefore, we conclude that inhibitory preBötC interneurons maintain inspiratory and sigh burst discharge behavior in pyr-3, which sustains the overall magnitude of preBötC field potentials even as drive potentials decrease in the Dbx1 preBötC subpopulation. While the activity of inhibitory interneurons affects phasic activity of respiratory microcircuits and sensorimotor integration [41,42,78,95], uniquely Dbx1-derived excitatory neurons of the preBötC and rVRG generate rhythm and drive phrenic premotor neurons [39] and thus directly impact inspiratory breathing movements, which includes sighs.

Koizumi and colleagues [45] reported a decrease in XII motor output and an increase in frequency (in some preparations, e.g., their Fig 4B, left). However, they applied pyr-3 at 50 μM, which exceeds the EC_50_ by 10-fold, so off-target effects on preBötC and XII motoneurons might be partly responsible. We also found that pyr-3 concentrations exceeding 10 μM diminished XII output and increased frequency (see S5 Fig), whereas there was no change in the XII output or frequency in response to 10 μM pyr-3, which still exceeds the EC_50_ [51,52].

A factor we cannot quantify is the extent to which the preBötC and ventral respiratory microcircuits compensate during the observation period to recover inspiratory and sigh burst-generating function in the face of Trpm4 (or Trpc3) knockdown [96]. Considering Trpm4 shRNA, which exerted the strongest effect, the cumulative effect of any compensatory changes were insufficient to rescue breathing: the effects on breath magnitude and frequency were comparable to acute effects of 9-phenanthrol in vitro from our data and those of Koizumi and colleagues [45].

### Trpm4-mediated *I*_CAN_ contributes to breathing predominantly in Dbx1 preBötC neurons

The preBötC contains roughly half excitatory neurons (Dbx1-derived) and half inhibitory neurons [41,54,97,98], but the shRNA does not discriminate neuron types. Dbx1 preBötC neurons are central for respiratory rhythm and motor output pattern in adult [34,35,37,99] and neonatal mice [32,33,36], so attenuating their activity would be expected to impact breathing. However, could Trpm4 knockdown in non-Dbx1 neurons explain, at least in part, the breathing phenotype here?

If Trpm4 shRNA reduced inspiratory burst magnitude in non-Dbx1 (presumably) inhibitory preBötC neurons, then it would increase (not decrease) breath size because it has been repeatedly demonstrated that disinhibition of the preBötC enhances breath size [41–43]. If we had observed an increase in breath size following Trpm4 (or Trpc3) shRNA, then it could be attributed to attenuation of *I*_CAN_ in non-Dbx1 inhibitory neurons of the preBötC, but breath size always decreased after shRNA. Furthermore, only 35% of inhibitory preBötC neurons express Trpm4 [45], and we show that 9-phenanthrol has no measurable effect on inspiratory and sigh bursts in glycinergic preBötC neurons. While we cannot conclude that shRNA knockdown in non-Dbx1 (presumably inhibitory) neurons has no effect on the preBötC, those effects are far less influential than shRNA knockdown in Dbx1 neurons, in which the preeminent role of Trpm4-mediated *I*_CAN_ is to magnify inspiratory bursts, which directly impacts breath size. Thus, we conclude that *Trpm4* knockdown reduces breath size by impeding *I*_CAN_-mediated burst generation in Dbx1 preBötC neurons.

### Ion channels differentiate rhythm and pattern generation in a canonical CPG

Breathing is a canonical behavior whose underlying rhythm and motor pattern emanate from a central pattern generator (CPG) network [100]. Whereas rhythm and pattern CPG functions are conjoint in lower vertebrates, mammalian motor behaviors incorporate multiple discrete phases (for locomotion there are multiple limbs and joints), which require CPGs with separable constituent parts: rhythmogenic cores that set the frequency and motor–pattern microcircuits that govern the amplitude and phase of the ensemble movements [101]. The breathing CPG shows us that pattern-related function encroaches on the rhythmogenic core.

Dbx1 preBötC neurons are inspiratory rhythmogenic [1], and some project to premotor and motor neurons [36,38,39,102]. Our data can be parsimoniously explained by Trpm4 (and to a lesser extent Trpc3) reduction affecting burst size, hence the number of spikes in Dbx1 preBötC neurons sent to premotor and motor neurons, which ultimately diminishes inspiratory breathing movements.

However, a suitable explanation for the breathing changes induced by Trpm4 (and to a lesser extent Trpc3) shRNA should consider the effects across the entire population of Dbx1 preBötC interneurons, not just those projecting to premotor or motor neurons. In that regard, “burstlet” theory may provide a framework for interpretation: it posits that recurrent synaptic excitation is sufficient for rhythm generation but subthreshold for motor output [5,6]. Burst generation largely attributable to Trpm4-mediated *I*_CAN_ plays a crucial role by propagating rhythmic activity from the preBötC core oscillator, i.e., Dbx1 interneurons that only connect to one another and can generate rhythms without high-amplitude bursts, to pattern-related and premotor microcircuits extrinsic to the preBötC. Thus, we identify discrete sets of ion channels, predominantly Trpm4 but to a lesser extent Trpc3, whose role in rhythmogenic interneurons pertains specifically to pattern generation. Therefore, rhythm and pattern generating functionality may, to some extent, be shared in canonical classes of CPG interneurons, a principle that may be generally applicable to understanding the neural bases of motor behavior in mammals.

## Materials and methods

### Ethics statement

The Institutional Animal Care and Use Committees (IACUC) at William & Mary and the Chicago Medical School of Rosalind Franklin University approved these animal protocols, which conform to the policies of the Office of Laboratory Animal Welfare (National Institutes of Health, Bethesda, MD, United States of America) and the guidelines of the National Research Council [103]. The specific protocols include IBC-2016-06-06-11257, IACUC-2018-05-01-12967, and IACUC-2016-08-02-11305 at William & Mary for CA Del Negro and B18-10 at Rosalind Franklin for K Kam.

For neuroanatomy or in vitro physiology experiments, neonatal mice were anesthetized by hypothermia and then killed by thoracic transection. Adult mice were killed via a lethal dose of pentobarbital (100 mg/kg body mass, IP). For in vivo injections, adult mice were anesthetized via ketamine (100 mg/kg, IP) and xylazine (10 mg/kg, IP).

### Animals

Mice were housed in colony cages on a 14-hour light/10-hour dark cycle with controlled humidity and temperature at 23°C, and were fed ad libitum on a normal rodent diet (Teklad Global Diets, Envigo, Madison, WI) with free access to water. At least three types of environmental enrichment materials were provided in each cage to improve the well-being of the mice.

For experiments pertaining to Dbx1 preBötC interneurons, we used knockin mice that express Cre recombinase fused to the tamoxifen-sensitive estrogen receptor *Dbx1*^*CreERT2*^ [104] (IMSR Cat# JAX:028131, RRID:IMSR_JAX:028131) and floxed reporter mice with inducible expression of the red fluorescent protein variant tdTomato dubbed Ai9 by the Allen Institute for Brain Science [105] (IMSR Cat# JAX:007905, RRID:IMSR_JAX:007905).

We crossed homozygous *Dbx1*^*CreERT2*^ females with Ai9 males. We refer to their offspring as Dbx1;Ai9 mice. To achieve optimal tdTomato expression in Dbx1-derived neurons, we administered a 22.5 mg/kg dose of tamoxifen (Sigma Aldrich, St. Louis, MO) dissolved at a concentration of 10 mg/ml in corn oil by oral gavage to pregnant dams at embryonic day 9.5 [47]. Newborn Dbx1;Ai9 pups express tdTomato in Dbx1-derived neurons in the preBötC and contiguous regions of the medulla [106].

For experiments pertaining to glycinergic preBötC interneurons, we used transgenic mice that express eGFP under the control of the promoter for the glycine transporter 2 gene (*slc6A5*), i.e., GlyT2-eGFP mice [53].

For anatomy or physiology experiments (see below), we used Dbx1;Ai9, GlyT2-eGFP, and wild-type CD-1 mice of both sexes at postnatal day 0 to 4 (P0-4). *Dbx1*^*CreERT2*^ mice have a CD-1 genetic background. Ai9 and GlyT2-eGFP mice have a C57Bl/6 background. The anatomy of respiratory networks is well documented in the brainstem of P0-4 Dbx1;Ai9 (CD-1 background) and C57Bl/6 mice [107,108], so the relative position of respiratory nuclei, particularly the preBötC, is stable during postnatal development (P0-4) across mouse strains.

Neonatal Dbx1;Ai9, GlyT2-eGFP, and wild-type CD-1 mice were anesthetized by hypothermia and then killed by thoracic transection. The neuraxis was removed in less than 2 min and further dissected in a dish filled with artificial cerebrospinal fluid (aCSF) containing (in mM): 124 NaCl, 3 KCl, 1.5 CaCl_2_, 1 MgSO_4_, 25 NaHCO_3_, 0.5 NaH_2_PO_4_, and 30 dextrose equilibrated with 95% O_2_-5% CO_2_, pH 7.4.

### Trpm4 and Trpc3 immunohistochemistry

The neuraxes of Dbx1;Ai9 pups were fixed in 4% paraformaldehyde (PFA) for 16–24 hours, rinsed in phosphate buffered saline (PBS) (BP399-1, Fisher Scientific, Hampton, NH), then immobilized in 4% agar for sectioning in the transverse plane. preBötC sections (50–100 μm thick) were incubated with 20% normal donkey serum (50-413-367, Fisher Scientific) overnight at 4°C on an orbital shaker. The following day, sections were washed with PBS 2 x 30 min and were then incubated overnight in PBS with 0.4% TritonX-100 at 4°C on an orbital shaker in the presence of primary antibodies. Those included (1) 1:250 rabbit polyclonal anti-Trpm4 corresponding to amino acids 60–74 (NH2-TEWNSDEHTTEKPTDC-COOH) of the amino-terminal tail of rat Trpm4 with an added carboxyl-terminal cysteine [109] whose specificity was tested via western blot of protein extracted from cultured MCF-7 cells [110] as well as western blot and single-cell RT-PCR in mouse brain tissue by the authors (R. Teruyama); (2) 1:500 rabbit polyclonal anti-Trpc3 (ACC-016, RRID:AB_2040236, Alomone Labs, Jerusalem) whose specificity was verified using a Trpc3 knockout mouse [111]; and (3) 1:500 goat polyclonal anti-GFAP (RRID:AB_880202, Abcam Cat #Ab53554, Abcam, Cambridge, MA) whose specificity was tested in mouse brain lysate using western blot (the antibody registry lists 14 different citations in rat and mouse). Sections were then washed 4 x 30 min with PBS and placed in a secondary antibody. Those included (1) 1:200 donkey anti-rabbit Alexa 647 (711-605-152, RRID:AB_2492288); (2) 1:200 donkey anti-rabbit Alexa 405 (711-475-152, RRID:AB_2340616); or (3) 1:200 donkey anti-goat Alexa 647 (705-605-147, RRID:AB_2340437). All secondaries were obtained from Jackson ImmunoResearch Labs (West Grove, PA). Sections were incubated in secondary antibodies for 1 hour at room temperature before being rinsed 6 x 30 min in PBS.

For immunohistochemistry in adults, Dbx1;Ai9 mice of both sexes all within the age range 10–14 weeks were transcardially perfused with PBS followed by 4% PFA. Neuraxes were removed and placed in 4% PFA overnight, rinsed with PBS, then immobilized in 4% agar for sectioning. Transverse preBötC sections (50–100 μm thick) were incubated in 1:250 rabbit anti-Trpm4, 1:400 rabbit anti-Trpc3, or 1:500 goat anti-GFAP in PBS with 0.4% TritonX-100 overnight at 4°C on an orbital shaker. Sections were then washed 2 x 30 min with PBS and incubated with 20% normal donkey serum with 0.4% TritonX-100 for 90 min on an orbital shaker at room temperature. Then, after two 10-min washes, the sections were placed in 1:200 donkey anti-rabbit Alexa 647, 1:100 donkey anti-rabbit Alexa 405, or 1:200 donkey anti-goat Alexa 647 for 1 hour at room temperature. Sections were then washed for a minimum of 6 x 30 min in PBS.

All sections were mounted on glass slides and cover-slipped, and images were acquired with a 10x air (NA 0.45), 40x water immersion (NA 1.15), and 60x oil immersion (NA 1.49) objectives on a confocal microscope (Nikon, Melville, NY) and (Thorlabs, Newton, NJ).

### shRNA constructs

Specific shRNA sequences designed to knockdown *Trp* transcripts (CCTAACTCACTGATCCGAAAT for Trpm4, NCBI 68667, RefSeq NM_175130.4, and GAGGTTCAATATTTCACCTATGC for Trpc3, NCBI 22065, RefSeq NM_019510.2) [112] were incorporated into a viral vector, which features a U6 polymerase III promoter to drive shRNA expression and a CMV promoter to drive eGFP expression for identification of transduced neurons (Cyagen Biosciences, Santa Clara, CA). We chose an AAV vector with serotype 9 to package the shRNA because AAV9 achieves high transduction of neurons, but not microglia or oligodendrocytes, in mouse brains [113]. We also used a nontargeting shRNA (CCTAAGGTTAAGTCGCCCTCG), which is a randomized sequence from *Trpm4* but has no homology to any known genes in mouse or human as a control. The standard titer of AAV is ≥1 x 10^13^ GC/ml (genome copies per ml).

### In vivo AAV injections

Our group has been studying respiratory function of Dbx1-derived neurons for nine years using the *Dbx1*^*CreERT2*^ mouse strain with a CD-1 background [37,91,114–119]. Therefore, we used CD-1 mice for in vivo behavioral experiments. CD-1 mice of both sexes, all within the age range of 10–14 weeks, were anesthetized via ketamine (100 mg/kg, IP) and xylazine (10 mg/kg, IP) and positioned in a stereotaxic frame. We exposed the skull aseptically and drilled two holes with coordinates 6.9 to 7.8 mm caudal to bregma, 1.2 mm lateral to midline on both sides, at a dorsal-ventral depth of 5.2 mm from the brain surface. We injected 100 nl of virus into each hemisphere through a 30-gauge 2.0 μl Hamilton Neurosyringe (Hamilton Company, Reno, NV) at rate of 50 nl/min. The needle was left in place for an additional 5 min to allow for diffusion. The scalp was then stapled, and triple antibiotic cream containing neomycin, bacitracin, and polymyxin antibiotics was applied to the wound. The mice recovered on a heating pad and were closely monitored postoperatively for signs of infection, pain, or distress. Local antibiotics and ketoprofen (5 mg/kg) was administered on an as-needed basis. We periodically measured breathing behavior for 6 weeks before killing the mice to examine the extent of virus transduction and expression. Following transcardial perfusion and fixation, transverse sections of the medulla (75 μm thick) were examined for eGFP expression in the preBötC. The area of virus expression for all mice was quantified by superimposing confocal sections from individual mice onto corresponding sections from a mouse atlas [120]. Pixel intensity in unprocessed 12-bit confocal microscopy sections (4,096 values) was equally divided into low (0–2,048) and high (2,049–4,096) intensity levels. Low-intensity contours were plotted at 10% transparency; high-intensity contours were plotted at 20% transparency. The darkest area of coloration indicates the center of virus expression, while lightly colored areas indicating peripheral regions with lower expression.

### ddPCR to measure Trp knockdown

In separate experiments to assess the effectiveness of shRNA knockdown of *Trp* transcripts, adult CD-1 mice of both sexes, all within the age range of 10–14 weeks, were injected with 200 nl of AAV containing either Trpm4-targeted shRNA or Trpc3-targeted shRNA in the left preBötC and a nontargeting control sequence in the right preBötC. Three animals were tested (i.e., three biological replicates) in each group targeting either *Trpm4* or *Trpc3* (*n* = 6 mice total). Each animal served as its own control because the left preBötC was injected with either Trpm4 or Trpc3 shRNA, and the right preBötC was injected with control shRNA containing a randomized sequence without a known gene target.

After 5 weeks of incubation, the adult mice were rapidly killed, and the bilateral halves of the preBötC region were isolated and separated to extract RNA that was reversed transcribed to cDNA and then processed to quantify gene expression. Briefly, total RNA was extracted using RNAzol RT (RN 190, Molecular Research, Cincinnati, OH) and further purified with 4-bromoanisole (BAN) to eliminate contaminating DNA. RNA was normalized to 500 ng across all samples and then reversed transcribed to cDNA using the iScript Reverse Transcription Supermix for RT-qPCR (1708840, Bio-Rad, Hercules, CA). RNA and cDNA concentration and quality were measured using NanoDrop One Microvolume UV-VIS Spectrophotometer (ND-ONE-W, Thermo Fisher Scientific, Waltham, MA). We used a final concentration of 2.5 ng/μl cDNA to quantify *Trpm4* and *Trpc3* via droplet digital PCR (i.e., ddPCR) (QX100, Bio-Rad), which partitions samples into thousands of droplets then amplified by PCR using Taqman Gene Expression Assays (*Trpm4*: TCTTGTGAAAGCCTGTGGGAGCTCT, Mm00613173_m1, RefSeq NM_175130.4; or *Trpc3*: CCTTGTAGCAGGCTGGGGAAGATTC, Mm00444690_m1, RefSeq NM_019510.2; 4331182, Life Technologies, Carlsbad, CA). The hydrolysis primers and probes in the Taqman assays were designed to span exon–exon junctions to avoid amplifying the target from genomic DNA, if present. Following PCR amplification, the Quantasoft software v1.7.4 (Bio-Rad) analyzed the number of positive (containing at least one copy of the target) and negative droplets. Absolute copy number of transcripts per μl of cDNA was determined by fitting the fraction of positive droplets to a Poisson distribution. A no-template control and a negative control from the reverse transcription reaction of selected samples were also included in the ddPCR assay, which returned a zero count, as expected.

### Western blot to analyze protein knockdown

Adult CD-1 mice of both sexes, all within the age range of 10–14 weeks, were injected with 100 nl of AAV containing either Trpm4-targeted shRNA (*n* = 12) or Trpc3-targeted shRNA (*n* = 12) in the left preBötC and a nontargeting control sequence in the right preBötC. Three mice from each group were rapidly killed at each time point (day 10, 20, 30, or 40 postinjection), and the bilateral halves of the preBötC region were dissected for protein extraction. Brain tissues containing the preBötC were homogenized and sonicated in 500 μl cold lysis buffer (Cell Signaling Technology, Danvers, MA) containing 1 mM protease inhibitor phenylmethylsulfonyl fluoride (PMSF) added immediately before use. After centrifugation of brain lysates at 16,000 x g for 15 min, crude total protein concentrations of the supernatants were determined using Nanodrop One Spectrophotometer A280 analysis (Thermo Fisher Scientific).

Approximately 80 μg of total protein was resolved in 4%–12% gradient gels (NP0321, Thermo Fisher Scientific) and transferred onto polyvinylidene difluoride membrane. After blocking with OneBlock Western (20–314, Genesee Scientific, San Diego, CA) for 1 hour at room temperature, membranes were probed with primary antibodies against either Trpm4 (1:200) or Trpc3 (1:250) and the loading control beta-actin (1:1000, Abcam Cat# ab8224, RRID:AB 449644), followed by 1:15,000 polyclonal secondary antibodies IRDye 680RD goat anti-rabbit (LI-COR Biosciences Cat# 925–68071, RRID:AB_2721181) or IRDye 800CW goat anti-mouse (LI-COR Biosciences Cat# P/N 925–32210, RRID:AB_2687825). Both primary and secondary antibodies were diluted in the blocking buffer. Washes after primary and secondary antibody incubations were done with 1x TBS with 0.1% Tween-20 for a minimum of 3 x 10 min. Immunoblot signals were captured and quantified using Odyssey CLx Infrared Imaging System (Li-Cor Biosciences, Lincoln, NE).

### Breathing measurements: Whole-body plethysmography

We measured breathing behavior in unrestrained, awake adult CD-1 mice of both sexes, all within the age range of 10–14 weeks (*n* = 26 mice total), using a whole-body plethysmograph (EMKA Technologies, Falls Church, VA) with a flow rate of 1 l/min in normoxia (21% O_2_ and 79% N_2_). The airflow traces were analyzed using the spirometry module in LabChart 7 software (AD Instruments, Colorado Springs, CO). We measured V_T_, T_I_, and breathing rate (frequency). MV was calculated by multiplying the V_T_ and frequency. The mice were introduced to the plethysmograph 1 week prior to AAV injection via three 30-min sessions inside the chamber, the first with a lightly closed but unsealed chamber, and the final two sessions with a sealed chamber and balanced airflow conditions. Those acclimatization sessions were not analyzed. For analyses, we performed plethysmography before shRNA injection (day 0), which we define as baseline breathing, and then intermittently for 6 weeks. Mice were recorded in 2 continuous 30-min sessions every 6 days. The day of each recording session is indicated by x-axis in Fig 6. The mice were placed in the sealed chamber with balanced air flow 10 min prior to data collection during each session for acclimatization. Human experimenters observed the mice firsthand during every session. The mice were alert to their environment, which is consistent with wakefulness. Locomotion, grooming, and sniffing (with synchronized whisking) entrain and modify breathing [1,121–123], so we only analyzed epochs of calm breathing absent other orofacial or motor behaviors (e.g., Fig 4). Epochs of calm breathing represent eupnea. The sum of the duration of epochs of eupnea always exceeded 2 min for each 30 min session. Sighs, which are periodic large magnitude augmented inspiratory efforts [106], could be distinguished by inhaled volume exceeding V_T_ by 2–3-fold (the inspired air during a sigh draws on the “inspiratory reserve volume” of the lungs, and thus exceeds V_T_ by definition). We excluded sighs from the analyses whenever they were embedded within an epoch of eupnea. Excluding sighs was important because of their large volume and because of the prolonged pause that follows each sigh; both of which confound measurements of V_T_, T_I_, and frequency. Gasps can be distinguished from sighs based on their rise time being approximately half as long and because an active exhale precedes a gasp. We also excluded gasps from analyses of eupnea.

We nonetheless counted the number of sighs within the 30-min recording period to compute sigh frequency. However, we could not accurately compute sigh volume or duration because sighs occurred during all behavioral states (locomotion, grooming, sniffing, as well as eupnea) and attempting to measure sigh volume and duration in other behavioral states yields inaccuracies due to fluctuations that obscure functional residual capacity, i.e., equilibrium lung volume and variable respiratory demand. There is no MV pertaining to sighs because they are episodic events interspersed among eupneic breaths, not continuous.

### Breathing-related measurements in vitro: Brainstem slices and electrophysiology

Isolated neuraxes from Dbx1;Ai9, GlyT2-eGFP, and CD-1 wild-type neonatal mice (0–4) of both sexes were fixed to an agar block and then cut in the transverse plane to obtain a single 550-μm–thick slice that exposes the preBötC at its rostral face [106]. Slices were then perfused with aCSF at 28°C in a recording chamber on a fixed-stage microscope with epifluorescence to visually identify tdTomato- or eGFP-expressing target neurons. Extracellular K^+^ was increased to 9 mM to elevate preBötC excitability [72].

Inspiratory-related motor output was recorded from the XII, which are captured in transverse slices, using suction electrodes and a differential amplifier. We also simultaneously recorded field potentials from the preBötC. Amplifier gain was set at 2,000, and the band-pass filter was set at 300–1,000 Hz. XII and preBötC bursts were full-wave rectified and smoothed for display.

We obtained whole-cell patch-clamp recordings under visual control using pipettes with resistance of 4–6 MΩ and a Dagan IX2-700 current-clamp amplifier (Minneapolis, MN), an EPC-10 patch-clamp amplifier in current-clamp mode (HEKA Instruments, Holliston, MA), or a Molecular Devices Multiclamp 700B patch-clamp amplifier in current clamp mode (San Jose, CA). All recordings were digitally acquired at 4 kHz using after 1 kHz low-pass filtering.

The patch-pipette solution contained (in mM) 140 K-Gluconate, 10 HEPES, 5 NaCl, 1 MgCl_2_, 0.1 EGTA, 2 Mg-ATP, and 0.3 Na(3)-GTP. We added 50 μM of Alexa 488 hydrazide dye (A10436, Life Technologies) to the patch solution for nonred fluorescent visualization of Dbx1 preBötC interneurons recorded in the whole-cell configuration.

We measured the area and amplitude of preBötC field potentials and inspiratory drive potentials, respectively, after digital smoothing. In the case of inspiratory drive potentials, smoothing eliminates spikes but preserves the underlying envelope of depolarization of the inspiratory drive potential [124]. We also measured the amplitude and area of low-frequency sighs (<0.01 Hz), i.e., augmented bursts with two peaks (doublet) that typically have a greater amplitude and often double the area of normal inspiratory bursts [73–75].

### Pharmacology

The Trpm4 antagonist 9-phenanthrol [50] (211281, Sigma) (10, 50, 100, and 200 μM) and Trpc3 antagonist pyrazole-3 (pyr-3) [51,52] (P0032, Sigma) (1, 10, and 50 µM) were bath-applied while monitoring field potentials in the preBötC and XII motor output. We applied BAPTA (30 mM), FFA (100 μM), and 9-phenanthrol (100 μM) via dialysis through the patch pipette to test their effects on individual Dbx1 preBötC neurons without attendant network effects. Pyr-3 (10 μM) is only effective when applied extracellularly [51,52].

### Statistics

We plotted V_T_, frequency, T_I_, and MV versus day post-shRNA injection and then performed linear regression analyses to obtain the best-fit slope (Prism, Graphpad Software, La Jolla, CA). Each individual measurement, rather than the mean value for each day post-shRNA, was considered a separate point in the analysis. We report the goodness of fit (r^2^) along with the 95% confidence interval. We applied an F test to evaluate the slope of the relationships of V_T_, frequency, T_I_, and MV versus day post-shRNA. Assuming there is no relationship, i.e., slope is zero, then the F statistic returns a *P* value quantifying the likelihood of obtaining a slope deviating from zero. We used orthodox criteria (alpha of 0.05) to judge whether a slope was significantly nonzero and thus that a trend exists for V_T_, frequency, T_I_, or MV versus day post-shRNA. For the electrophysiology data, we used paired *t* tests to compare measurements between control and drug. Data are reported as mean ± SD.

## Supporting information

S1 Fig Trpm4 expression in neonatal preBötC neurons. tdTomato (Dbx1, magenta) and Trpm4 (cyan) expression in preBötC neurons from a Dbx1;Ai9 mouse aged postnatal day 1. Trpm4 is expressed in both Dbx1 neurons (arrowheads) and non-Dbx1 neurons (arrows). Dbx1, developing brain homeobox 1; preBötC, pre-Bötzinger complex; Trp, transient receptor potential.
(TIF)

S2 Fig Trpc3 expression in neonatal preBötC neurons. tdTomato (Dbx1, magenta) and Trpc3 (cyan) expression in preBötC neurons from a Dbx1;Ai9 mouse aged postnatal day 1. Trpc3 is expressed in both Dbx1 neurons (arrowheads) and non-Dbx1 neurons (arrows). Dbx1, developing brain homeobox 1; preBötC, pre-Bötzinger complex; Trp, transient receptor potential.
(TIF)

S3 Fig Western blot analysis of Trpm4 and Trpc3 expression in the preBötC of adult CD-1 mice injected with Trpm4- or Trpc3-shRNA and NT shRNA at several time points postinjection. All six lanes from each timepoint consist of three biological replicates (A, B, and C) taken from the same blot. Beta actin served as a loading control. Fluorescence signal representing protein abundance is plotted at right for all four time points (mean [magenta] is shown with bars for SD). Primary data can be found in the Supporting information (S1 Data). NT, nontargeting; preBötC, pre-Bötzinger complex; shRNA, short hairpin RNA; Trp, transient receptor potential.
(TIF)

S4 Fig shRNA-eGFP expression in the preBötC. Viral expression in the preBötC and ventral medulla superimposed on transverse sections adapted from an adult mouse atlas [120]. Top row (−6.59 mm from bregma) is rostral to the preBötC at the level of the BötC, IRt, and NAc. Middle row (−6.95) is at the level of the preBötC, NAsc, and IO. Bottom row is at the level of the rVRG, LRN, and the external (a.k.a., loose) division of the NAe. Viral expression is shown for cohorts injected with Trpm4 shRNA (left, cyan), Trpc3 shRNA (center, magenta), or nontargeting (control) shRNA (right, gray). Overlapping shaded contours reflect the level of viral expression across animal subjects. The sections measure 4.25 mm in width, 2.1 mm in height. eGFP, enhanced green fluorescent protein; IO, inferior olive; IRt, intermediate reticular formation; LRN, lateral reticular nucleus; NAc, compact division of the nucleus ambiguus; NAe, nucleus ambiguus; NAsc, semicompact division of the nucleus ambiguus; preBötC, pre-Bötzinger complex; rVRG, rostral ventral respiratory group; shRNA, short hairpin RNA; Trp, transient receptor potential.
(TIF)

S5 Fig Dose-response experiments for 9-phenanthrol and pyr-3. Typical slice recording of preBötC field potential and XII motor output (top). Group data for preBötC inspiratory burst area (black, upper plot), XII output (gray, upper plot), and frequency (black, lower plot) in response to increasing concentrations of 9-phenanthrol (left) and pyr-3 (right) in neonatal CD-1 mice. The abscissae are plotted logarithmically but the concentrations are not log-transformed. Primary data can be found in the Supporting information (S1 Data). preBötC, pre-Bötzinger complex; pyr-3, pyrazole-3; XII, hypoglossal nerve root.
(TIF)

S6 Fig Trpm4 and Trpc3 antagonists attenuate inspiratory and sigh bursts of Dbx1 preBötC neurons. Each row shows whole-cell recordings at the onset of recording (t0, left column) and at steady-state (ss, right column). These records correspond to the bursts shown in Fig 8 but at a slower sweep speed to show more cycles. Here the synchronous XII output is also shown (gray) for each condition. The rows, from top to bottom, show control (no drug dialysis), BAPTA, FFA, 9-phenanthrol, and pyr-3 (the same order as Fig 8). Primary data can be found in the Supporting information (S1 Data). BAPTA, (1,2-bis(2-aminophenoxy)ethane-*N*,*N*,*N′*,*N′*-tetraacetic acid); FFA, flufenamic acid; preBötC, pre-Bötzinger complex; pyr-3, pyrazole-3; Trp, transient receptor potential.
(EPS)

S7 Fig Trpm4 and Trpc3 antagonists do not attenuate inspiratory or sigh bursts in glycinergic preBötC neurons. Group data quantifying inspiratory (left) and sigh (right) bursts at the onset of whole-cell recording (t0) and at ss (following approximately 20 min of drug exposure) for both Trp channel antagonists, 9-phenanthrol (top row) and pyr-3 (bottom row). All of these data are from neonatal GlyT2-eGFP mice. Means and SD bars (magenta) are shown with raw data from each glycinergic neuron recorded. We detected no significant effects. Primary data can be found in the Supporting information (S1 Data). GlyT2, glycine transporter 2; eGFP, enhanced green fluorescent protein; preBötC, pre-Bötzinger complex; pyr-3, pyrazole-3; ss, steady-state; Trp, transient receptor potential.
(EPS)

S1 Data Primary dataset. This file contains relevant numerical data used to generate figures in this manuscript.
(XLSX)

S1 Checklist ARRIVE checklist. ARRIVE, Animal Research: Reporting In Vivo Experiments.
(PDF)

## Abbreviations

AAV: adeno-associated virus
BAPTA: (1,2-bis(2-aminophenoxy)ethane-*N*,*N*,*N*',*N*'-tetraacetic acid)
Ca^2+^: calcium
CPG: central pattern generator
Dbx1: developing brain homeobox 1
EC_50_: half-maximal effective concentration
eGFP: enhanced green fluorescent protein
FFA: flufenamic acid
*I*_CAN_: calcium-activated nonselective cationic current
MV: minute ventilation
NCBI: National Center for Biotechnology Information
PiCo: postinspiratory complex
preBötC: pre-Bötzinger complex
pS: picosiemens
pyr-3: pyrazole-3
RNA-seq: RNA sequencing
RPKM: reads per kilobase of transcript per million mapped reads
rVRG: rostral ventral respiratory group
shRNA: short hairpin RNA
T_I_: inspiratory time
Trp: transient receptor potential
V_T_: tidal volume
XII: hypoglossal nerve root
