# Supplementary figures and images for: Trpm4 ion channels in pre-Bötzinger complex interneurons are essential for breathing motor pattern but not rhythm
Source: PLoS Biol. 2019 Feb 21;17(2):e2006094. doi: 10.1371/journal.pbio.2006094 (PMC6400419; doi:10.1371/journal.pbio.2006094)

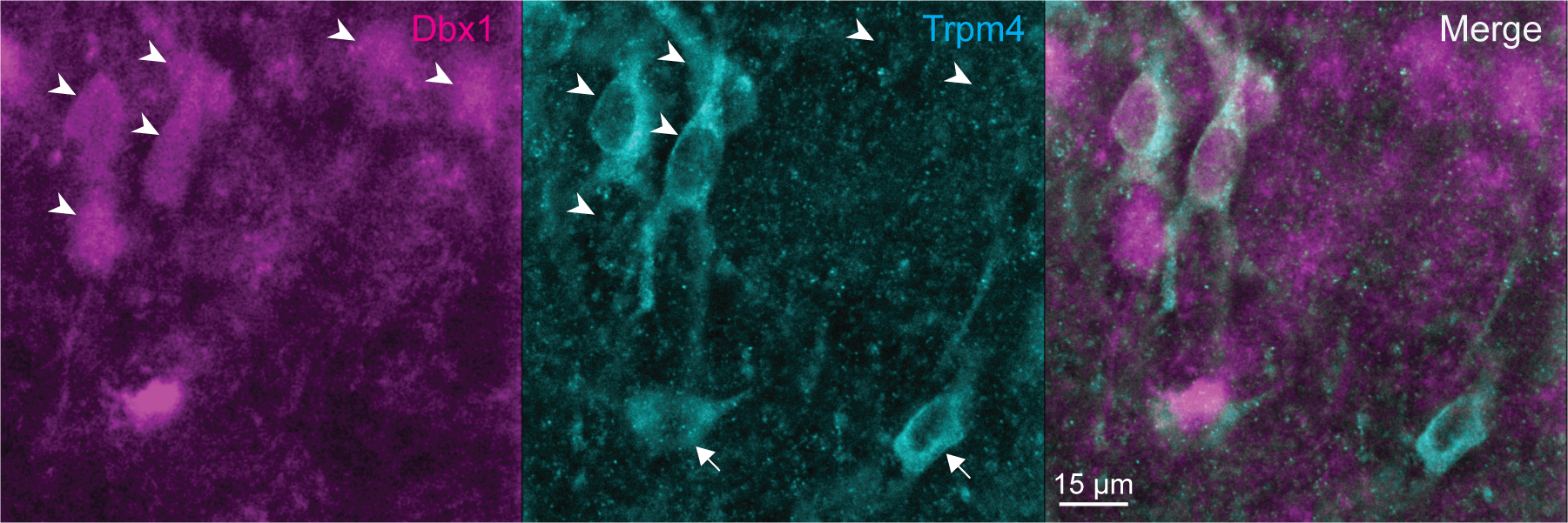

Supplement: S1 Fig — tdTomato (Dbx1, magenta) and Trpm4 (cyan) expression in preBötC neurons from a Dbx1;Ai9 mouse aged postnatal day 1. Trpm4 is expressed in both Dbx1 neurons (arrowheads) and non-Dbx1 neurons (arrows). Dbx1, developing brain homeobox 1; preBötC, pre-Bötzinger complex; Trp, transient receptor potential. (TIF) [file pbio.2006094.s001.tif]

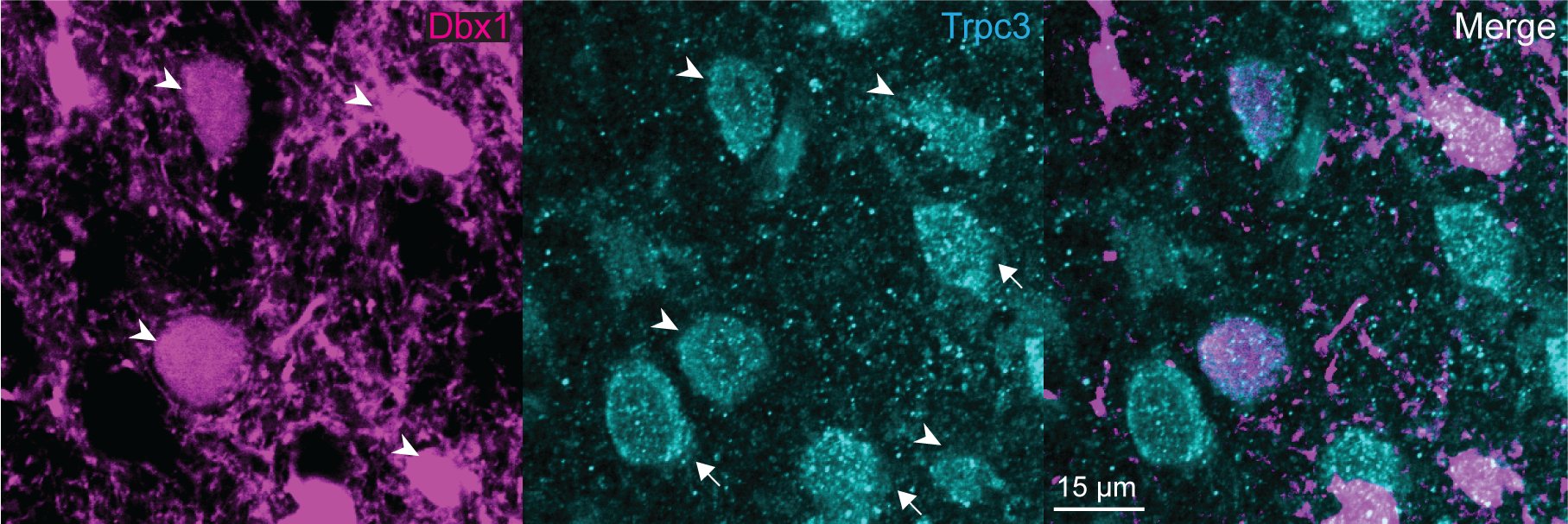

Supplement: S2 Fig — tdTomato (Dbx1, magenta) and Trpc3 (cyan) expression in preBötC neurons from a Dbx1;Ai9 mouse aged postnatal day 1. Trpc3 is expressed in both Dbx1 neurons (arrowheads) and non-Dbx1 neurons (arrows). Dbx1, developing brain homeobox 1; preBötC, pre-Bötzinger complex; Trp, transient receptor potential. (TIF) [file pbio.2006094.s002.tif]

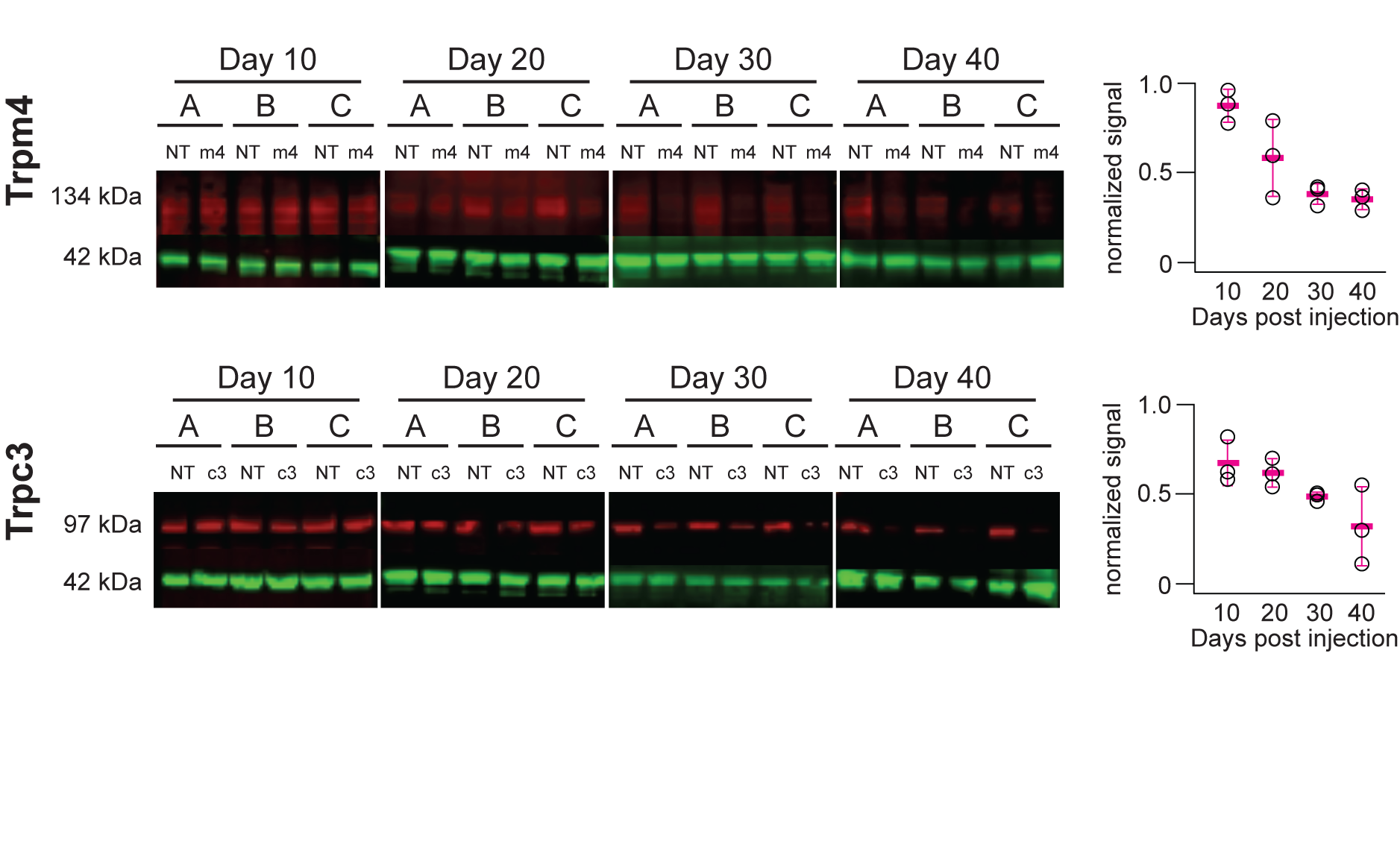

Supplement: S3 Fig — All six lanes from each timepoint consist of three biological replicates (A, B, and C) taken from the same blot. Beta actin served as a loading control. Fluorescence signal representing protein abundance is plotted at right for all four time points (mean [magenta] is shown with bars for SD). Primary data can be found in the Supporting information (S1 Data). NT, nontargeting; preBötC, pre-Bötzinger complex; shRNA, short hairpin RNA; Trp, transient receptor potential. (TIF) [file pbio.2006094.s003.tif]

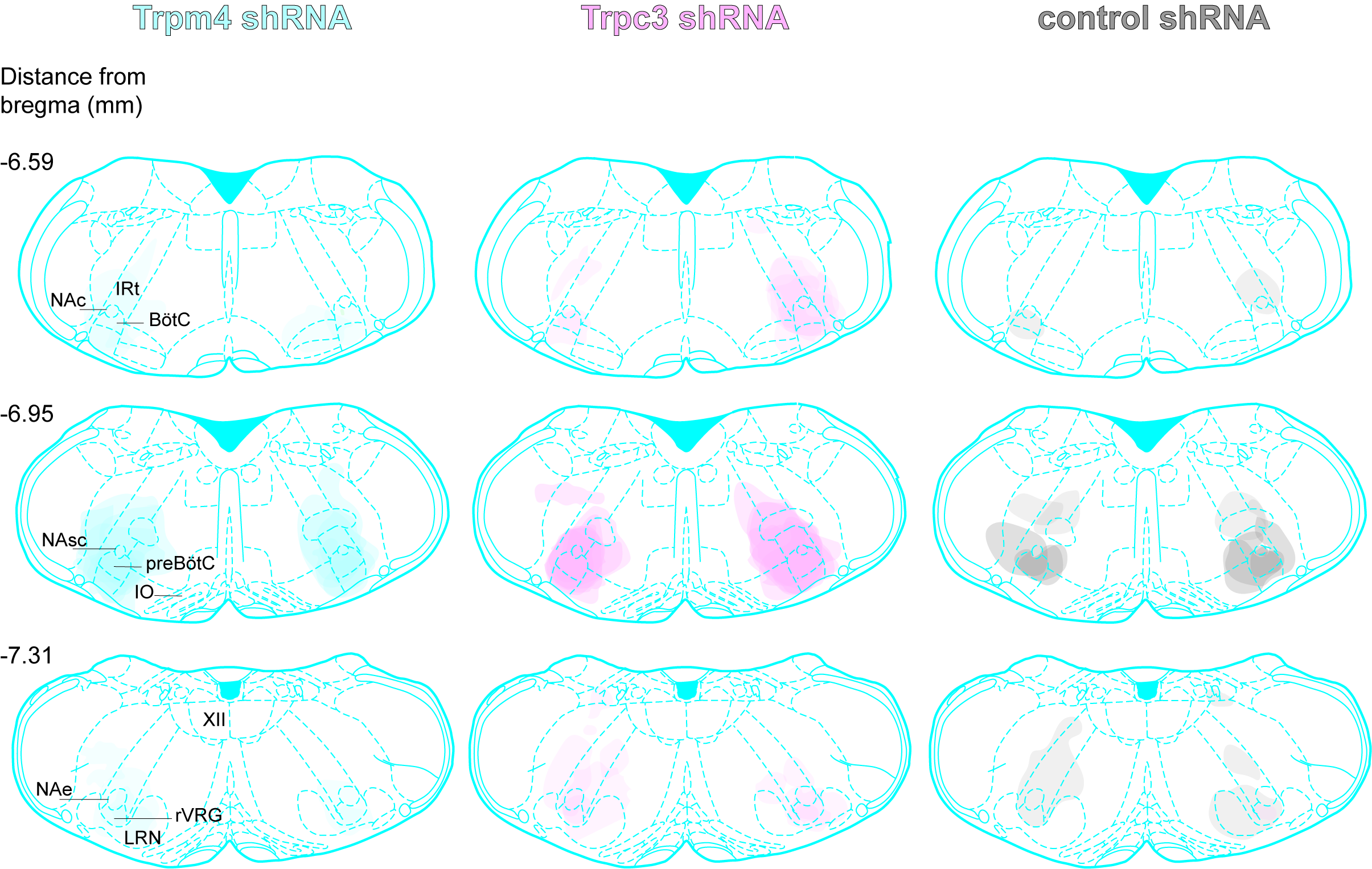

Supplement: S4 Fig — Viral expression in the preBötC and ventral medulla superimposed on transverse sections adapted from an adult mouse atlas [120]. Top row (−6.59 mm from bregma) is rostral to the preBötC at the level of the BötC, IRt, and NAc. Middle row (−6.95) is at the level of the preBötC, NAsc, and IO. Bottom row is at the level of the rVRG, LRN, and the external (a.k.a., loose) division of the NAe. Viral expression is shown for cohorts injected with Trpm4 shRNA (left, cyan), Trpc3 shRNA (center, magenta), or nontargeting (control) shRNA (right, gray). Overlapping shaded contours reflect the level of viral expression across animal subjects. The sections measure 4.25 mm in width, 2.1 mm in height. eGFP, enhanced green fluorescent protein; IO, inferior olive; IRt, intermediate reticular formation; LRN, lateral reticular nucleus; NAc, compact division of the nucleus ambiguus; NAe, nucleus ambiguus; NAsc, semicompact division of the nucleus ambiguus; preBötC, pre-Bötzinger complex; rVRG, rostral ventral respiratory group; shRNA, short hairpin RNA; Trp, transient receptor potential. (TIF) [file pbio.2006094.s004.tif]

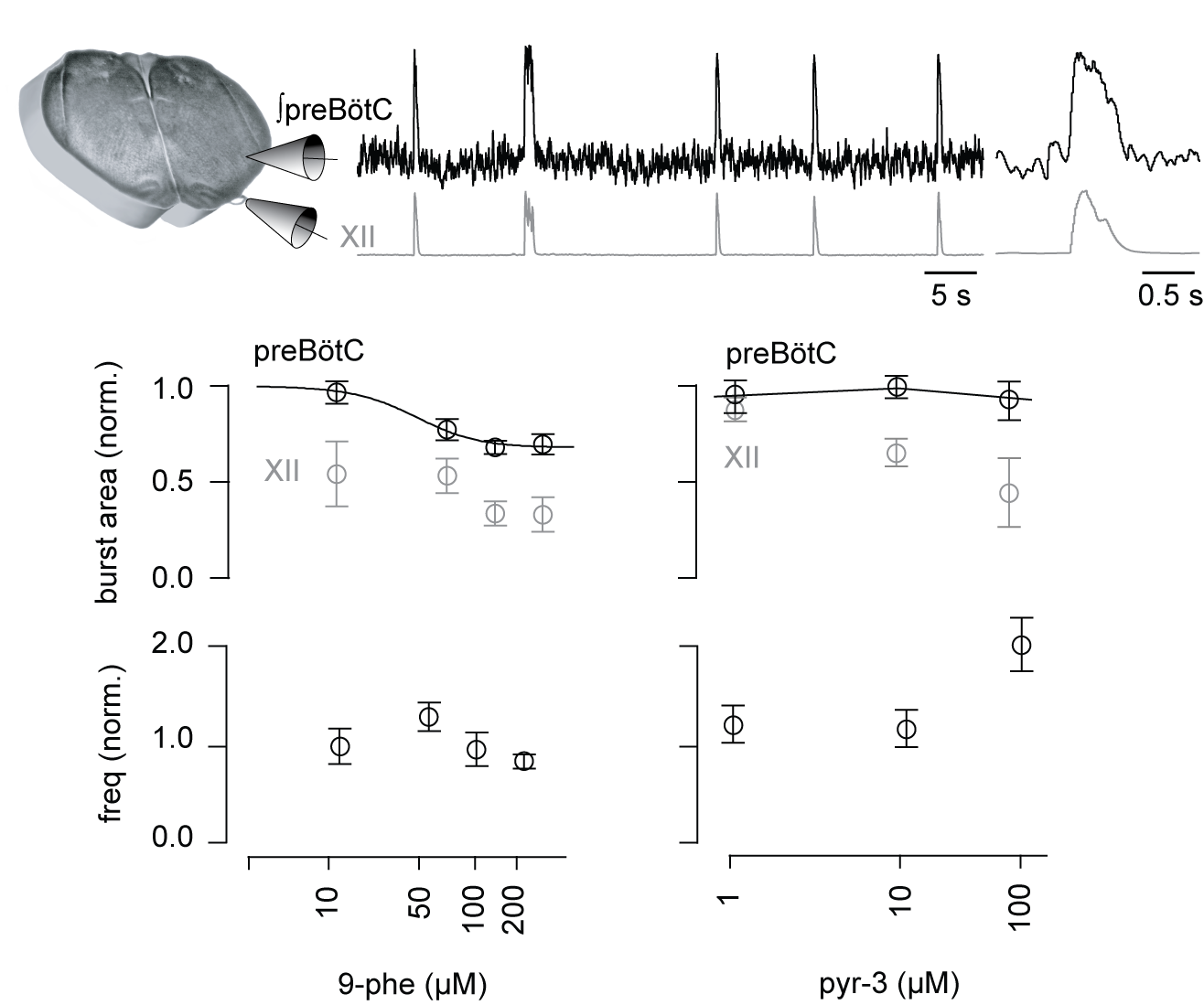

Supplement: S5 Fig — Typical slice recording of preBötC field potential and XII motor output (top). Group data for preBötC inspiratory burst area (black, upper plot), XII output (gray, upper plot), and frequency (black, lower plot) in response to increasing concentrations of 9-phenanthrol (left) and pyr-3 (right) in neonatal CD-1 mice. The abscissae are plotted logarithmically but the concentrations are not log-transformed. Primary data can be found in the Supporting information (S1 Data). preBötC, pre-Bötzinger complex; pyr-3, pyrazole-3; XII, hypoglossal nerve root. (TIF) [file pbio.2006094.s005.tif]
